# Behavioural, morphological and life-history adjustments in the early steps of colonising restricted semi-natural environments in wild house mice

**DOI:** 10.1186/s12862-026-02590-0

**Published:** 2026-09-24

**Authors:** Ekaterina Gorshkova, Daniela E. Winkler, Anja Guenther, Christine Böhmer

**Affiliations:** 1 Zoology and Functional Morphology of Vertebrates, Zoological Institute, Christian-Albrechts-Universität zu Kiel, Am Botanischen Garten 3–9, 24118 Kiel, Germany; 2 RG Behavioural Ecology of Individual Differences, Max Planck Institute for Evolutionary Biology, 24306 Plön, Germany; 3 Bonn Institute for Organismic Biology (BIOB), Department Paleontology, University of Bonn, Nussallee 8, 51331 Bonn, Germany; 4 Zoologie und Tierökologie, Universität Hildesheim, Universitätsplatz 1, 31141 Hildesheim, Germany

**Keywords:** Island syndrome, Colonisation, Risk-taking, Vertebral morphology, Life-history traits

## Abstract

**Background:**

Invasive species often exhibit rapid adjustment and establishment in novel environments. However, the mechanisms underlying this process remain poorly understood, particularly during the early stages of invasion. Understanding how behavioural, morphological, and life-history traits vary across generations under different environmental conditions is important for identifying the factors that contribute to invasion success. We investigated how different diet compositions influence these traits across multiple generations (F0-F3) of wild house mice in semi-natural enclosures, with one enclosure assigned to each dietary regime. By experimentally altering the composition of the diet, we aimed to determine the role of diet as one of the specific components of the Island syndrome, which is associated with rapid adjustment to a novel habitat.

**Results:**

Our results showed that stress-coping behaviour did not differ significantly across diets or generations. However, the F0 generation exhibited lower body weight than subsequent generations, sperm count was higher in this generation as well, suggesting an immediate physiological response to altered dietary conditions to help with population establishment. Survival rates remained stable across generations for mice on Hard and Soft but decreased for animals on Veggie diet.

**Conclusions:**

Overall, the findings suggest that early responses to invasion are driven more by phenotypic plasticity and life-history shifts, albeit not necessarily in an adaptive way.

**Supplementary Information:**

The online version contains supplementary material available at https://doi.org/10.1186/s12862-026-02590-0.

## Background

Understanding how species colonise new environments is of major interest in ecological and evolutionary research, particularly in light of the current global climate change. It is particularly unclear why certain species successfully establish and spread, sometimes reaching invasive status and causing severe ecological damage, while others do not. Little is known about the early steps of specific adjustments to novel environments, yet these initial changes are critical for survival and establishment [23]. The theoretical concept of the “Island Syndrome” (IS) [1] provides a robust framework for generating predictions about the rapid adjustment processes of species introduced to islands and island-like environments. It refers to a suite of predictable differences that can be observed in island populations/species compared with their mainland counterparts, driven by the unique selective pressures of island habitats [20]. These differences typically include an increase in body size for small animals, as well as behavioural changes such as increased risk-taking behaviour due to the absence of predation and/ or competition, and changes in life-history traits, specifically those related to reproductive effort [1, 4]. In this study, we examine the early steps of population establishment in the wild house mouse (*Mus musculus domesticus*) in semi-natural habitats with different dietary resources, each resembling an island-like habitat, being spatially confined, predator-free, and limited in diet diversity. House mouse populations can be considered island-like as their habitats (farms, shelters etc.) often appear as isolated patches within an otherwise unsuitable habitats such as forests or other natural habitats [40] house mice are well known for their invasive success, largely due to their close association with humans [67]. Using these experimental “island populations”, we investigate changes in behaviour, morphology, and life history over 4 generations to better understand the mechanisms underlying the early steps of invasion processes, focusing on the influence of diet.

**Fig. 1:**
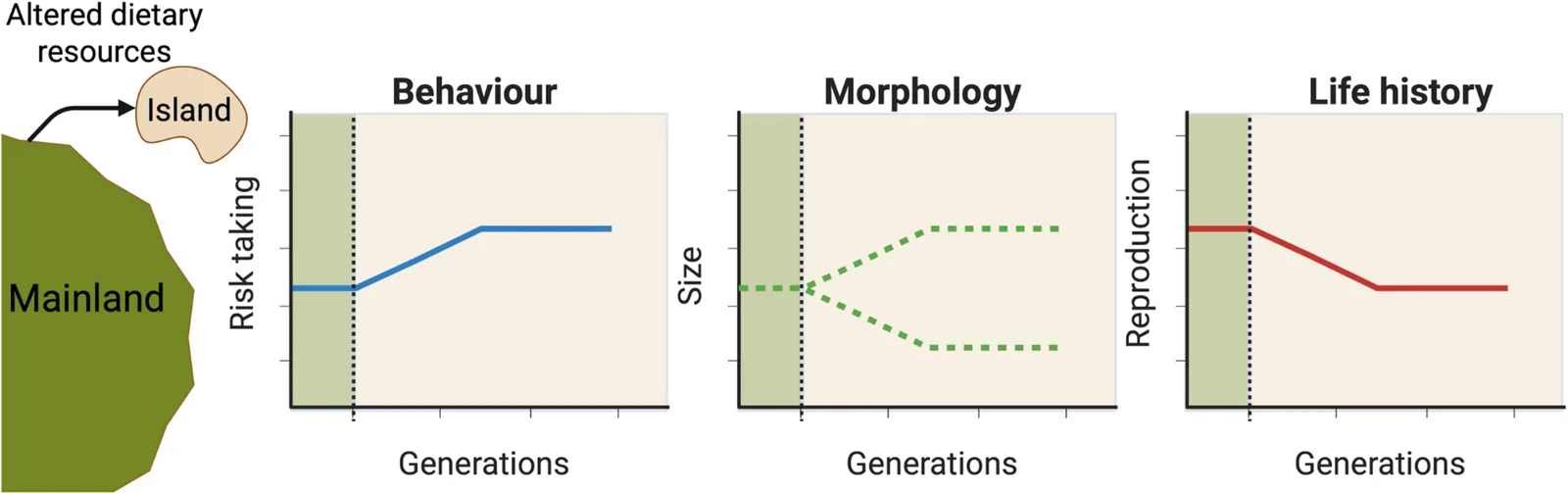
“Island syndrome” refers to a suite of predictable differences between island species and their mainland counterparts, driven by the unique selective pressures of island habitats. We hypothesise increased risk-taking behaviour, changes in body size and reduced reproduction in our experimental “island populations”

Islands may offer distinct dietary resources compared to the mainland habitat. Typically, island animals face diets of lower and more variable quality compared to those on the mainland [9, 33, 49]. These altered dietary resources (e.g. availability, quality or type of diet) can drive trait changes [32], potentially in conjunction with altered levels of intra- and inter-specific competition [18]. For instance, risk-taking behaviour is expected to change in accordance with predation risk on islands, alongside shifts in foraging strategies to cope with limited dietary resources [63]. Another pattern closely linked to dietary resource availability concerns body size. While small vertebrates on islands often become larger than their mainland counterparts, larger species tend to undergo dwarfing [42, 61].

Dietary availability and quality are well-established factors influencing behaviour, morphology and life history of animals [2, 10, 12, 29, 30, 56]. For example, individuals in better condition often behave risk-averse, while those in poorer conditions tend to take more risks to gain new resources or improve their environment [5, 16, 46]. While generally behavioural changes to island colonisation remain an underexplored aspect of the IS, dietary availability and quality are described as important factors shaping the behaviour of island taxa [22, 47]. Specifically, risk-taking is a key behaviour that helps animals survive and adapt to novel environments such as represented by islands [68]. Across several insular populations of rodents, higher levels of risk-taking have been reported compared to their mainland counterparts [35, 63]. However, we do not have information on the duration of population isolation or the timeline for the establishment of these island populations. When the timing of divergence is known, the findings suggest that behavioural changes can occur quickly in those invasive species. For example, a colonising population of round gobies (*Neogobius melanostomus*) was more risk-prone and dispersive compared to a longer established population [48]. On the other hand, in the invasive bank vole (*Myodes glareolus*), individuals that colonised within the last 1–4 years (equalling 1–12 generations), were more risk-averse yet more exploratory than long-established populations (> 80 years old) [19]. Together, the examples suggest that the ability to adjust behaviour in response to dietary availability and the condition of a novel environment is a key component of invasion success.

Morphological variation is strongly influenced by diet as well. The most commonly observed changes include changes in body proportions, skull shape, jaw structure and limb length. Prolonged undernutrition during prenatal and postnatal development significantly reduced skeletal growth in rats, with only partial recovery observed after refeeding [66]. Different diet compositions can affect cranial morphology; for example, ferrets raised on a soft diet exhibited reduced masticatory muscle function, resulting in a narrower face and less robust mandibular structures than those fed a hard diet [31]. In island environments, morphological changes are the most widely documented features of IS. Animals living on islands often develop distinct morphological traits that reflect the unique ecological pressures of island environments [1, 42, 68]. House mice (*Mus musculus*) on a Sub-Antarctic island, for example, exhibited rapid evolutionary changes in the shape of their skulls and mandibles within 16 years [57]. In addition, the population of house mouse (*Mus musculus*) on Gough Island in the South-Atlantic Ocean, roughly 3000 km south of Kapstadt, have also experienced a significant increase in body mass since their introduction to the island [25, 34], accompanied by an overall increase in skeletal size and changes in shape [50].

A key component of life history is reproductive investment. It is usually traded off against growth and therefore body size, and is another aspect along which IS may manifest, as living on islands often requires animals to adjust how and when they reproduce [9, 68]. Accordingly, many island rodents tend to have smaller litters and delayed sexual maturity compared to their mainland counterparts. This pattern has been documented in many rodents, such as island deer mice (*Peromyscus maniculatus*) and island wood mice (*Apodemus sylvaticus*) [1]. Accompanying physiological changes can be observed. For example, cane toad (*Rhinella marina*) males from the invasion front had smaller testes than males from a long-established population, suggesting lower sperm competition and lower investment into reproduction [21]. Besides reproduction, survival is also affected by the IS. Prolonged growth phases, delayed reproduction and reduced investment in reproduction can only be advantageous if individuals survive long enough to have reproductive success later in life [58]. This idea is supported by observations on *Podarcis* lizards, which indicate that insular populations exhibit longer lifespans than those on the mainland [59] and an insular population of Virginia opossums (*Didelphis virginiana*), showing higher survival rates compared to mainland populations [3]. Nevertheless, we still lack information on how these reproductive changes affect the invasion success of newly established populations.

Whilst the majority of studies on IS have focused on populations in which the syndrome is already established (i.e., several hundred-thousand generations after the colonisation event), it is important to consider the initial experiences and adjustment processes of the first generation of relocants who, for various reasons, settled in these isolated environments. We still know little about how animals respond in the first generations after colonising isolated, novel habitats. In the present study, we derived experimental “island populations” from an established semi-natural population of wild house mice (*Mus musculus domesticus*). This semi-natural population was part of an experiment investigating the effects of changes in diet quality (caloric intake) across multiple generations and traits. Dietary-induced adjustments of life-history, behaviour and underlying gene expression were observed within three generations [44, 54]. Fastest adjustments were observed in life history traits (fecundity rates, growth and generation times), which already showed significant differences within the first two generations. Adjustments in risk-taking behaviour, measured as the distance covered in an open field but also as aggression, followed in the third generation, although short-term behavioural adjustments directly following a change in diet were also observed. The same dietary regimes induced differences in male courtship and aggressive behaviour, albeit not in sperm counts or testes to body mass ratios [53]. Using one of the populations that never experienced a change in diet for 6 generations as a founder (mainland) population, we here simulated artificial island environments (size-limited semi-natural environments) with different diet compositions but similar caloric intakes. By experimentally changing the diet of several successive generations of wild house mice, we aimed to determine the role of diet as one of the specific factors contributing to the IS associated with rapid adjustment to a novel habitat. This helps us to test IS predictions under controlled conditions, which is nearly impossible on real islands or invasion fronts where factors such as predation, competition, or parasitism may influence the outcome. Thus, we introduced mice into the novel environments and followed them until the populations were established over three generations. However, it should be noted that we do not fully simulate true islands, but rather only selected aspects associated with the colonisation of the island habitat. Across generations, we compared their behavioural, life-history, and morphological traits with those of the mainland mice, which had remained under stable conditions without relocation or dietary changes. Specifically, we examined differences in boldness and stress responses (i.e., rapid behavio-physiological reactions that occur when an animal perceives a threat), both aspects of how animals take risks, as well as growth patterns, skeletal size (vertebral length and count), sperm count, and survival, compared with the mainland control group. Based on described effects for rodents in Fig. 1, we predict that the founder mice would show increased risk-taking behaviour following relocation and diet change, larger body size in the following generations, and reduced reproductive investment. According to previous studies manipulating diet quality rather than diet composition, we expect changes in risk-taking and life history to emerge within the first 1–2 generations, induced by phenotypic plasticity rather than changes in gene frequency [54]. Such early responses may arise through phenotypic plasticity, which allows individuals to adjust their phenotype in response to novel environmental conditions. Phenotypic plasticity is the ability of an organism or genotype to consistently respond to environmental conditions. These responses can occur during development, across generations through parental influences, or during adulthood which is often referred to as phenotypic flexibility.

## Materials and methods

### Housing conditions

Commensal house mice are frequently present on farms, which represent islands of suitable habitat within a matrix of non-commensal habitat unsuitable for house mice due to the presence of more competitive small mammals [40]. On farms, the suitable habitat is patchily distributed, and mice tend to live in high-density populations at the scale of a few metres (e.g. stores of food) [8, 40]. They form a tight, demic structure with family groups defended by a dominant male and low dispersal between demes [6, 15, 26, 51]. Studying house mouse populations in semi-natural enclosures is comparable to the natural conditions of commensal house mice living in barns [37, 51, 52].

Our founder source/mainland semi-natural population was established using descendants of wild house mice (*Mus musculus domesticus*) originating from the Cologne/Bonn region in Germany (50°45ʹN–51°N, 6°45ʹE–7°E). All mice used in the experiment were drawn from a single population of mice from a six-generation semi-natural enclosure experiment. These individuals were young adults when released into “island” environments. A total of 210 founding mice were used, of these, 45 mice (21 females and 24 males) were kept in their original enclosure without any dietary change or relocation and served as the control group (ML). The remaining 165 mice formed F0 generations as they were divided into three groups and transferred to new semi-natural enclosures, each assigned a specific dietary regime with caloric content comparable to the control diet (“Hard”, “Soft”, “Veggie”) (for timeline see Fig. S1). Thus each dietary treatment was represented by a single semi-natural enclosure, and the descendant generations were born within that enclosure, F0 mice were young adults of 5–7 weeks, an age in which physiological sexual maturity might be reached but animals usually start reproducing at the age of 3–4 months only [17]. Thus, the animals had no previous reproductive experience at the time of release into the “island” environments. Each newly transferred “island” population (i.e., each enclosure) initially consisted of 20 females and 20 males. To reduce potential inbreeding, individuals were allocated such that each enclosure contained at most one male and one female from each family. Semi-natural enclosures (*N* = 4; size = 19.6 m^2^) were designed to resemble the natural environment of house mice, i.e., a barn or another human shelter [36]. Each enclosure contained twelve nest boxes, wood chips, various nesting materials, and nine food and water stations (see [54] for details). Temperature and photoperiod followed natural seasonal patterns, though winter temperatures were maintained above 10 °C. To prevent overcrowding, we removed older animals from the enclosure as soon as the population exceeded 140 individuals, the carrying capacity of our enclosures [39]. From the mice that were removed, we immediately dissected males and females for dissection and analyses of sperm count (males) and pregnancy status (females), for sample sizes per generation and enclosure see Supplementary Tables 1 and 2.

### Diets

ML mice were fed pellet (“Altromin 1324”) containing 3227 kcal/kg of metabolizable energy (24% from protein, 11% from fat, and 65% from carbohydrate). Island diets were based on two grain mixtures that differ in the texture (Table S3). The “Hard” diet contained 25% broken peas, 22% wheat, 22% crushed barley, 21% oats, and 10% black sunflower seeds, and supplemented with commercial dry cat food mix (“Brekies”, RuCat-Premium 3-Mix). The “Soft” and “Veggie” diets had the same base consisted of 25% pea flakes, 22% wheat flakes, 22% barley flakes, 21% oat flakes, and 10% dehusked sunflower seeds. “Soft” diet was supplemented with black soldier fly larvae, whereas “Veggie” diet was supplemented with vegetable mixture (pea flakes, lucerne cobs, carrot flakes, potato cubes, parsnip cubes, beetroot cubes, and leek). All diets were composed to be comparable in caloric content while differing in mechanical properties and composition. Non-pregnant individuals consume an average of 6.62 g of standard laboratory mouse chow (ML food [54]) per 24 h. In contrast, they eat more of the natural foods provided here (Hard = 12.08, Soft = 8.1, Veggie = 11.13), with no difference between the diets offered (all *p* > 0.1) (Table S4).

### Stress coping in the Open Field

To capture mice from semi-natural environments, forty live traps were placed into an enclosure for 4–5 h, starting, depending on sunset timing, during evening twilight hours. Traps were controlled every 10–15 min, and mice that were not used for experiments were released immediately. We used 25 mice per treatment in each generation, except in the F2 generation, where no behaviour measurements were taken due to time-constraints. All Open Field (OF) tests were performed on adult mice aged 3–5 months. Mice tested in OF were transferred to the testing room in the trap and directly released into the OF. The OF is an open and unfamiliar space where animals are exposed to potential predators, so measurements with OF reflect how animals deal with perceived stress and risk [28]. Measurements taken in the open field have been validated for their ecological meaning in several rodent species. For example, the distance covered in the OF, reflects its exploration behaviour of large-scaled, semi-natural enclosures [38, 39]. The OF consists of a 60 × 60 cm^2^ white box with 70 cm-high walls to prevent mice from jumping out. For five minutes, we recorded the time spent in the central zone (the inner 30 cm square) of the OF, mobility percentage and the distance moved during this time using automatic tracking software (EthoVision). Animals from the correct generation were selected randomly when they were caught during trapping and tested for their OF response once. As individuals should be representative of the whole population, sex, age, body mass and time of testing were not controlled for. To measure direct physiological response to the OF, we took 3 thermal images of the mice before releasing the animal into OF and 3 thermal images right after using a FLIR T 860 infra-red camera. Increase in temperature in the eye-region is strongly correlated to an increase in neuro-physiological stress markers [41]. Thereafter, mice were immediately released back into the enclosure.

### Structural size determination by X-ray imaging

Following the sacrifice of the animals using CO_2_, whole-body X-rays were obtained from up to 16 males and up to 20 females per treatment group in each generation using a Faxitron Bioptics LLC Vision scanner (version 2.2.3). All of the animals used for the vertebral measurements had reached skeletal maturity (no epiphyseal plates). Individuals from F0 to F2 were of a similar age, while F3 individuals were slightly younger, but had also reached skeletal maturity by the time of measurement. Images were captured with an exposure time of 6 s and a tube voltage of 50–60 kV. Each specimen was positioned ventrally to obtain dorsoventral images of the vertebral column, which were saved in DICOM format. These files were then analysed using ImageJ (version 1.54 g) to count total number of vertebrae and to measure the centrum lengths of representative vertebrae in the cervical, lumbar, and caudal regions (C2, L1 and Ca1 (see Fig S2). Each measurement was repeated three times, and mean values were used in the final analysis.

### Reproduction

#### Sperm count

After humane sacrifice using CO_2_, the testes and epididymides were immediately dissected. For analysis, we obtained up to 16 males per treatment per generation. The left cauda epididymis was collected for sperm counting, and the testis mass of both testes, corrected for body mass (i.e., the Gonadosomal-Index), was measured. Samples for sperm count were weighed and placed in 1 mL of 1× phosphate buffer solution (PBS). The sperm suspension was then incubated in a thermomixer at 40 °C for 10 min to kill the sperm. Depending on sperm concentration, samples were diluted 1:10, 1:20, or 1:40 with 1× PBS to obtain an appropriate number of sperm for counting in a Bürker chamber. A 10 µL aliquot was loaded into the chamber and examined under a light microscope at 40× magnification (Ph2 filter). Sperm were counted in 25 squares (counting volume 0.1 µl), including damaged cells. Each sample was counted twice, and the mean of the two counts was used for analysis. The final sperm count was calculated by corresponding dilution factor.

#### First reproduction

During monthly monitoring of each semi-natural enclosure, pregnancy status for females was recorded, allowing identification of the first pregnancy in each female’s lifetime.

### Growth rate and survival

To record growth and survival rates, we conducted monthly monitorings of each semi-natural enclosure. During these checks, we temporarily removed all individuals from the enclosure to measure body mass. Mice weighing more than 10 g that were captured for the first time were implanted with a subcutaneous Radio-frequency Identification microchip, registered as new individuals, and added to the population database. Dead animals detected during monitorings were recorded and removed, providing monthly survival information.

### Statistical analysis

All statistical analyses were performed in R (version 4.4.1) [55]. All OF measurements were analysed using linear models fitted with the lm function. Covered distance and Time in the centre were analysed separately as response variables, with treatment included as a fixed effect. For vertebral measurements, linear models were used, with C2, L1, and Ca1 centrum length included as the response variable. Experimental group (defined by treatment and generation) and sex were included as fixed effects, body weight was included as a covariate to account for differences in body size. If the model indicated significant main effects, post hoc pairwise comparisons between groups were estimated using Tukey’s adjustment using emmeans package in R [60]. To measure growth rate, a linear mixed-effects model was fitted using the lmer function, with experimental group (defined by diet and population) and month included as fixed effects and individual included as a random effect [7]. Sperm count and testis mass were divided by body weight to account for differences in body size. Sperm count by body mass and testes by body mass ratios were analysed using linear models fitted with the lm function, with the experimental group included as a fixed effect. To determine differences in the age of first reproduction, the lm function was used, with age of first reproduction as the response variable and group as a fixed effect. Survival analysis was performed using Cox proportional hazards regression from survival package [64]. Survival time was defined as the number of months lived. Only natural deaths were included in this analysis. Survival status was coded as a binary variable, with individuals who died of natural causes coded as 1 and individuals removed/dissected coded as 0. Sex and experimental group were included as fixed effect, with ML population as the reference group.

## Results

### Behavioural differences

**Fig. 2:**
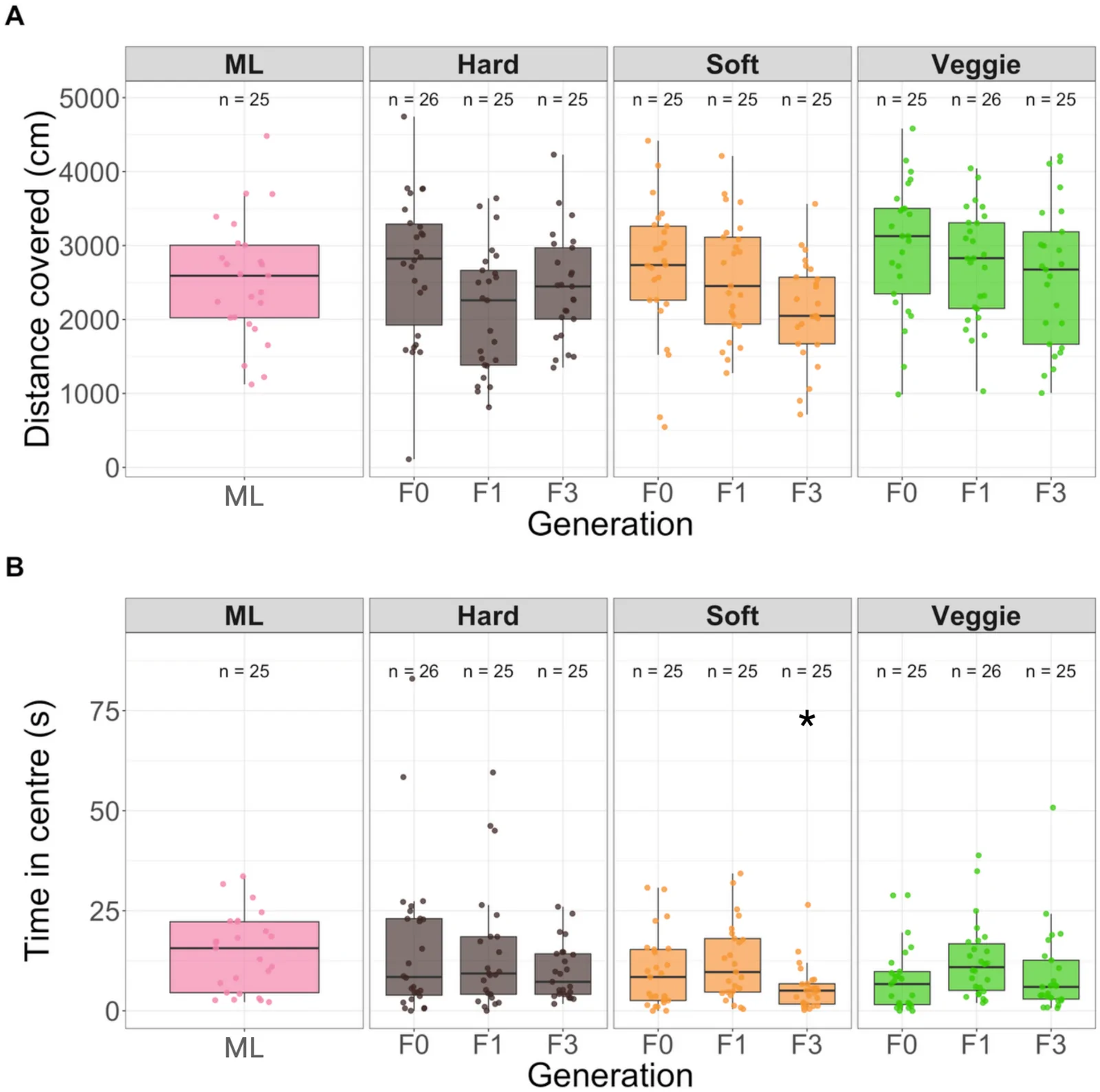
Open Field measurements for stress coping and risk-taking across generations (F0-F3) of “island” mice (Hard, Soft, Veggie) compared to the control group (ML): (**A**) Distance covered, (**B**) Time in centre. *Indicates significant divergence from ML

We examined behavioural differences between “island” mice and the control group (ML) using the OF test. We measured total covered distance to determine differences in mice’s stress-coping strategies. However, our findings indicate no differences in either measurement relative to control mice (ML) (Fig. 2, Table S5). The only exception was the F0 generation of mice on the Veggie diet, which showed reduced mobility (*p* = 0.031) (Table S4), i.e., mice were more passive stress-copers. Regarding risk-taking, we measured time spent in the centre; we again observed no significant divergence from the control (ML) group, the only exception was mice on a Soft diet from the F3 generation, which spent less time in the centre (*p* = 0.043) (Fig. 2, Table S5). We also measured body temperature changes from before and after the OF to assess the direct physiological response. Across all treatments and generations, the body temperature increased by average of 0.4 °C, and this increase was similar to that of the control population (ML) (Table S5). Overall, these findings indicate that no directed divergence in stress-coping from the control population appeared in the first three generations.

### Morphological differences

**Fig. 3:**
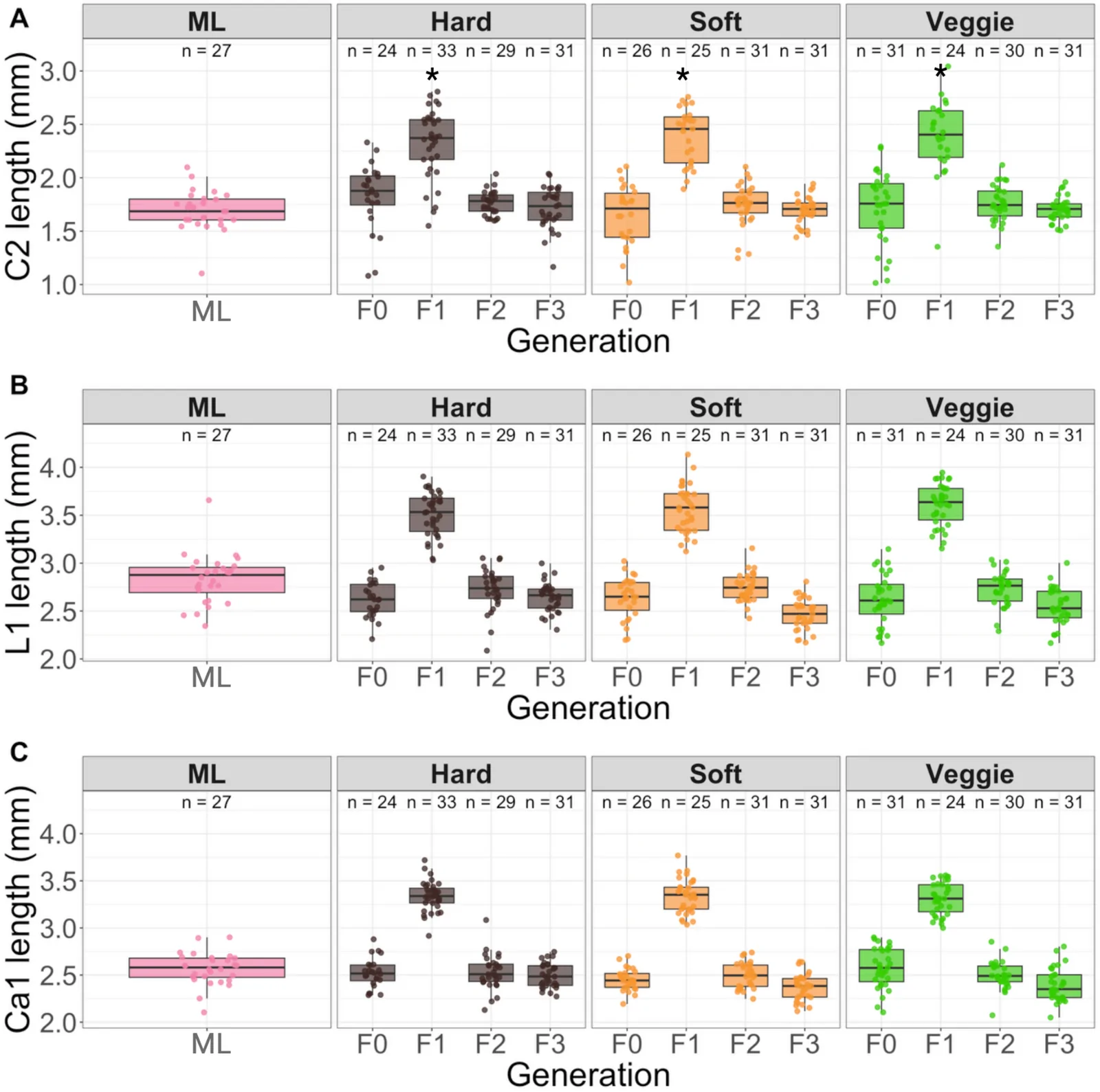
Differences in vertebral length across treatments and generations: (**A**) Second cervical vertebrae (C2) length, (**B**) First lumbar vertebrae (L1) length, (**C**) First caudal vertebrae (Ca1) length. *Indicates significant divergence from ML

The number of vertebrae counted up to the caudal region did not differ significantly between groups, except for mice on a Veggie diet in the F0 generation, where we observed a significantly reduced number of vertebrae (*p* < 0.001), however vertebral number is determined during development and cannot reasonably be interpreted as a response to the Veggie diet. We observed significant differences in the number of vertebrae in the caudal region (Table S6). This pattern may be influenced by the high amount of tail damage in males, which likely affected the outcome. Males frequently engage in aggressive interactions (territory defence and intra-sexual competition), making tail injuries and damage a well-known phenomenon. When we compared the lengths of the selected cervical, lumbar, and caudal vertebrae (here C2, L1, and Ca1), we found a significant divergence (Fig. 3; Table 1). In particular, the F1 generation of mice in all “island” treatments exhibited greater vertebral length than control mice (ML) and than the other generations within the same treatment. In addition, the length of L1 appears more plastic, as it differs significantly across generations within each treatment (Table 1).

**Table 1:** Post-hoc Tukey comparisons for the second cervical (C2), first lumbar (L1), and first caudal (Ca1) vertebrae (only significant results shown)

Vertebrae	contrast	estimate	SE	t.ratio	*p*.value
C2	F6 ML- F1_Hard	-0.62	0.06	-10.28	< 0.0001
C2	F6 ML- F1_Soft	-0.66	0.06	-10.28	< 0.0001
C2	F6 ML- F1_Veggie	-0.71	0.07	-10.94	< 0.0001
C2	F0_Hard - F1_Hard	-0.44	0.06	-6.93	< 0.0001
C2	F0_Soft - F1_Soft	-0.65	0.07	-9.78	< 0.0001
C2	F0_Veggie - F1_Veggie	-0.66	0.06	-10.40	< 0.0001
L1	F0_Soft - F3_Soft	0.17919	0.0506	3.543	0.0258
L1	F1_Soft - F2_Soft	0.84753	0.0481	17.616	< 0.0001
L1	F1_Soft - F3_Soft	1.00146	0.0495	20.231	< 0.0001
L1	F0_Veggie - F1_Veggie	-0.95765	0.0478	-20.014	< 0.0001
L1	F1_Veggie - F2_Veggie	0.87963	0.0481	18.287	< 0.0001
L1	F1_Veggie - F3_Veggie	1.05284	0.0477	22.088	< 0.0001
L1	F2_Veggie - F3_Veggie	0.17321	0.0492	3.523	0.0276
Ca1	F0_Veggie - F1_Veggie	-0.74	0.04	-18.42	< 0.0001
Ca1	F0_Veggie - F3_Veggie	0.18	0.04	4.44	< 0.0001

### Life history differences

**Fig. 4:**
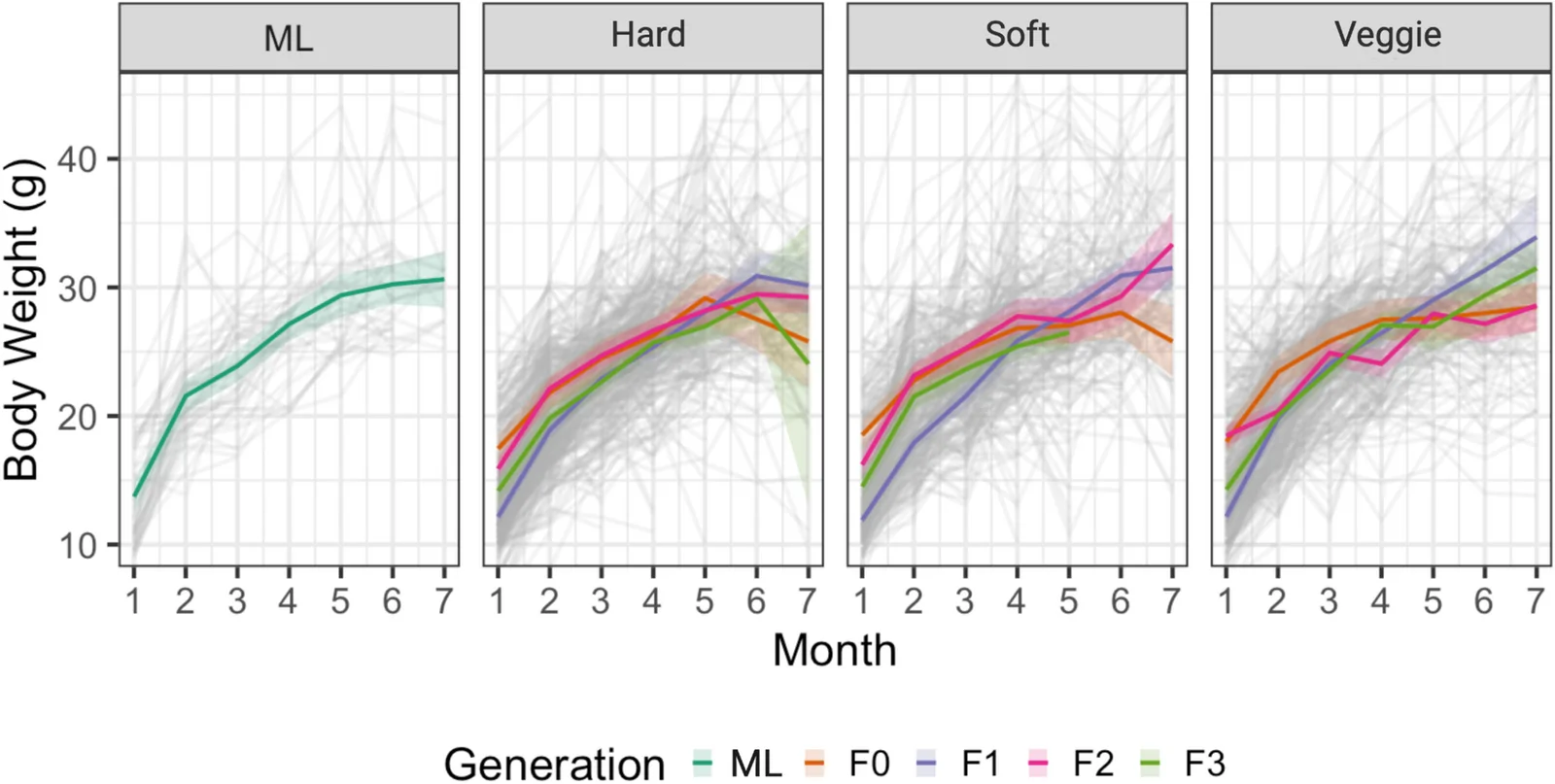
Monthly body mass development of mice across treatments and generations. Month indicate the age of the mice in months

To determine the differences in the development of body mass between the groups, we compared the treatment/generation combinations with the control group (ML) (Fig. 4). Our results showed that mice that experienced relocation (F0) exhibited significantly lower body masses than control mice (Table 2). However, by generation F1, no significant differences remained and growth rates were comparable with those of the control group. In the F2 generation, mice on Hard and Soft diets were again similar to the ML diet mice, but mice on a Veggie diet showed a significantly lower growth rate. By generation F3, there was no significant difference in growth rate across diets.

**Table 2:** Pairwise contrasts of specific growth rate for all Treatment × Generation combinations compared with the control group, significant comparisons are shown in bold

Contrast	Estimate	SE	z-ratio	*p*-value
**Hard.F0 − ML.F6**	**-0.08081**	**0.0198**	**-4.074**	**0.0005**
**Soft.F0 − ML.F6**	**-0.09329**	**0.0194**	**-4.812**	**< 0.0001**
**Veggie.F0 − ML.F6**	**-0.06869**	**0.0190**	**-3.606**	**0.0034**
Hard.F1 − ML.F6	0.00589	0.0162	0.363	0.9982
Soft.F1 − ML.F6	0.00878	0.0164	0.536	0.9907
Veggie.F1 − ML.F6	0.01292	0.0174	0.743	0.9653
Hard.F2 − ML.F6	-0.01621	0.0167	-0.971	0.9054
Soft.F2 − ML.F6	-0.01620	0.0171	-0.949	0.9127
**Veggie.F2 − ML.F6**	**-0.06234**	**0.0169**	**-3.680**	**0.0026**
Hard.F3 − ML.F6	-0.00692	0.0172	-0.401	0.9972
Soft.F3 − ML.F6	-0.04548	0.0187	-2.430	0.1272
Veggie.F3 − ML.F6	-0.00481	0.0176	-0.274	0.9995

### Reproduction

**Fig. 5:**
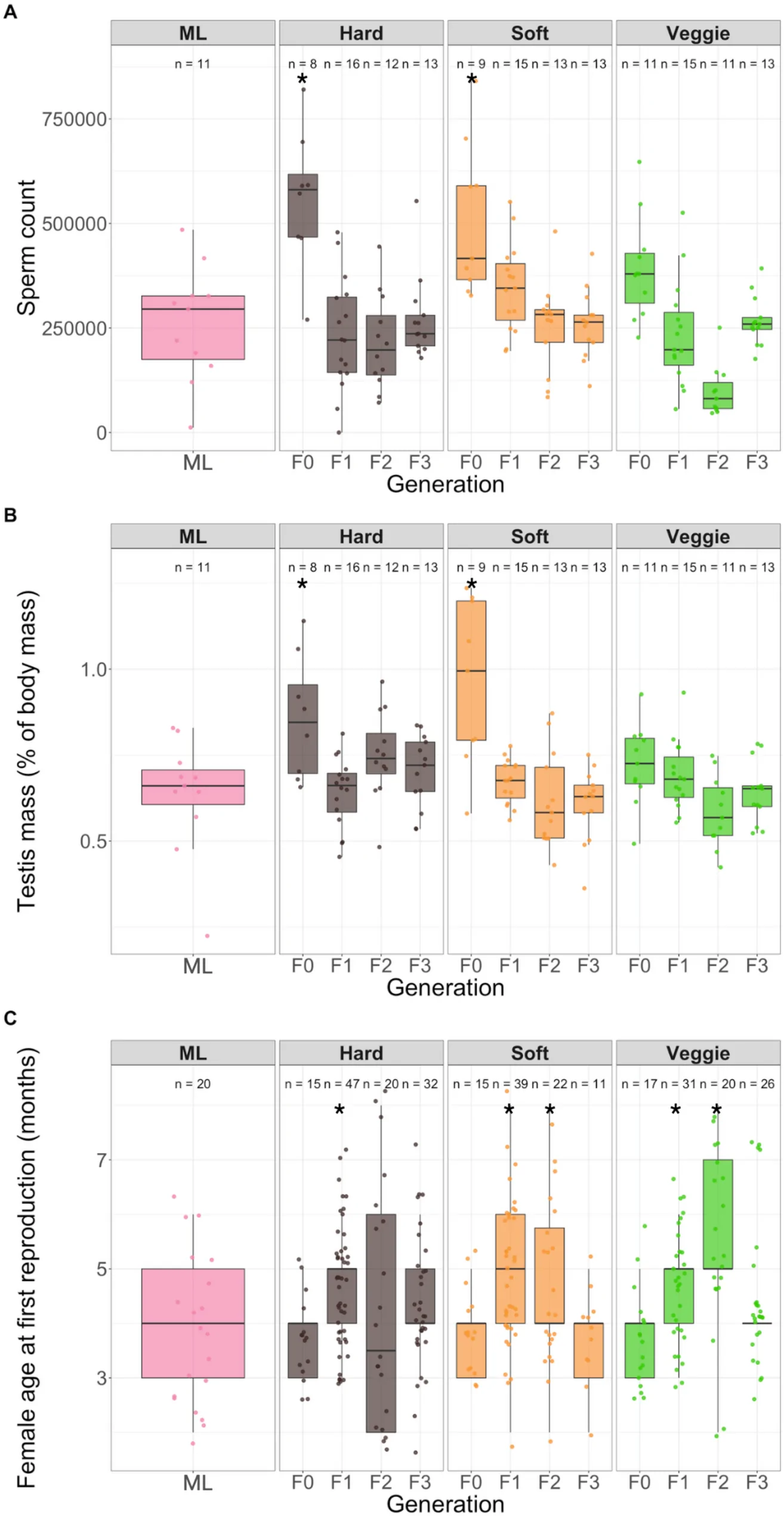
Differences in reproductive traits of mice: (**A**) sperm count, (**B**) testis mass as percentage of the body mass, (**C**) female age at first reproduction. *Indicates significant divergence from ML

Compared with control mice (ML), the F0 generation of mice on a Hard and Soft diet produced significantly more sperm. Furthermore, all F0 generation mice produced more sperm than subsequent generations (Fig. 5A; Table 3). Besides that, we also observed a similar pattern in terms of testes mass: mice in the F0 generation on a Hard and Soft diet had heavier testes than subsequent generations (Fig. 5B; Table 3).

**Table 3:** Pairwise contrasts between treatment groups for sperm count and testis mass (only significant results shown)

variable	contrast	estimate	SE	t.ratio	*p*.value
Sperm	F6_ML - F0_Hard	-298743.6	54065.34	-5.526	< 0.0001
Sperm	F6_ML - F0_Soft	-246626.3	52297.49	-4.716	0.0004
Sperm	F0_Hard - F1_Hard	325247.1	50383.01	6.455	< 0.0001
Sperm	F0_Hard - F2_Hard	344087.9	53108.36	6.479	< 0.0001
Sperm	F0_Hard - F3_Hard	288426.5	52284.92	5.516	< 0.0001
Sperm	F0_Soft - F2_Soft	251300.2	50454.73	4.981	0.0001
Sperm	F0_Soft - F3_Soft	248692.9	50454.73	4.929	0.0002
Sperm	F0_Veggie - F1_Veggie	156160.2	46187.90	3.381	0.0482
Sperm	F0_Veggie - F2_Veggie	292255.8	49613.76	5.891	< 0.0001
Sperm	F2_Veggie - F3_Veggie	-167709.9	47667.36	-3.518	0.0318
Testis	F6_ML - F0_Hard	-0.002226397	0.000584364	-3.810	0.0124
Testis	F6_ML - F0_Soft	-0.003261613	0.000565256	-5.770	< 0.0001
Testis	F0_Hard - F1_Hard	0.002135851	0.000544564	3.922	0.0084
Testis	F0_Soft - F1_Soft	0.002863004	0.000530257	5.399	< 0.0001
Testis	F0_Soft - F2_Soft	0.003428059	0.000545339	6.286	< 0.0001
Testis	F0_Soft - F3_Soft	0.003529883	0.000545339	6.473	< 0.0001

### Age of first reproduction

Difference in age at first reproduction in females is shown in Fig. 5C. Females from the F1 generation across all treatments tended to reproduce later than those maintained on a control treatment (Table 4). A similar delay was also observed in female mice from the Soft and Veggie diets in the F2 generation (Table 4). An interesting trend was also observed: all F1 generation mice reproduced later than F0 generation mice within the corresponding treatment (Table 4). Under a Veggie diet, F2 generation females also exhibit a delay in reproduction compared to F0 (*p* < 0.001).

**Table 4:** Results of the linear mixed effect model for age at the first reproduction (only significant results shown)

Contarst	Estimates	CI	*p*
F6_ML – F1_Hard	0.84	0.15–1.52	0.016
F6_ML – F1_Soft	1.05	0.34–1.75	0.004
F6_ML – F2_Soft	0.84	0.05–1.63	0.038
F6_ML – F1_Veggie	0.85	0.11–1.58	0.024
F6_ML – F2_Veggie	1.75	0.94–2.56	< 0.001
F0_Hard – F1_Hard	0.84	0.04–1.64	0.041
F0_Soft – F1_Soft	0.98	0.23–1.73	0.011
F0_Veggie – F1_Veggie	0.88	0.12–1.64	0.023
F0_Veggie – F2_Veggie	1.79	0.96–2.61	< 0.001

### Survival rate

**Fig. 6:**
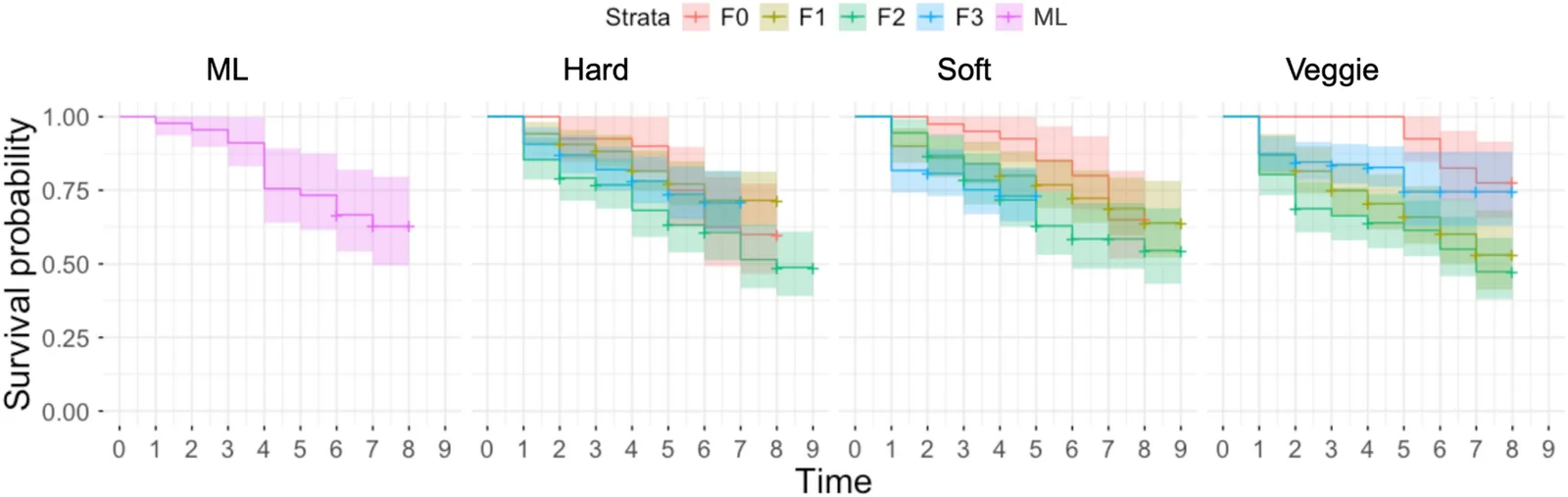
Estimates of survival probability over months. Number of animals died of natural causes per treatment and generation: ML F6–16, Hard F0–16, Hard F1–31, Hard F2–44, Hard F3–27, Soft F0–14, Soft F1–30, Soft F2–34, Soft F3–25, Veggie F0–9, Veggie F1–36, Veggie F2–52, Veggie F3–22

Compared with the ML population, no differences in survival were observed in the F0, F1, or F2 generations, however, all F3 mice showed significantly reduced survival compared to ML (Table 5; Fig. 6). Mice on the Hard and Soft diets showed no generational differences in survival relative to the F0 generation. In contrast, under the Veggie diet, F1 and F2 mice showed significantly reduced survival compared with F0.

**Table 5:** Cox proportional hazards models result for survival (only significant results shown)

Predictors	Estimates	CI	*p*
sex [m]	0.90	0.71–1.16	0.427
**Comparison with ML**			
group [F3.Hard]	2.01	1.08–3.75	**0.027**
group [F3.Soft]	3.80	1.99–7.23	**< 0.001**
group [F3.Veggie]	3.48	1.82–6.68	**< 0.001**
**Within diet comparison (F0 reference)**			
**Veggie**			
generation [F1]	2.77	1.33–5.79	**0.007**
generation [F2]	2.97	1.46–6.06	**0.003**

## Discussion

**Fig. 7:**
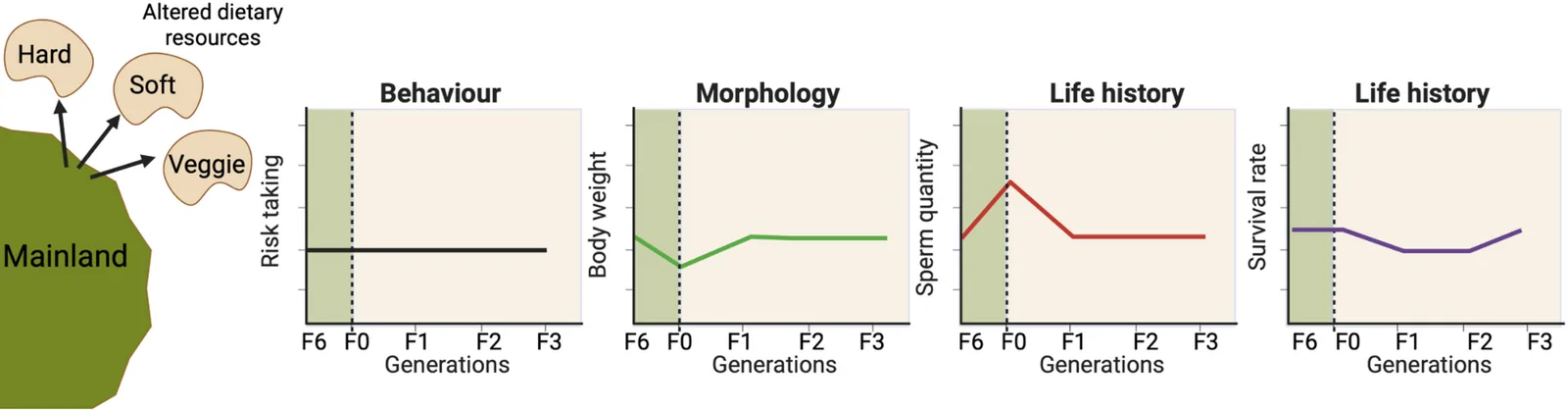
Changes in behaviour, morphology and life history across generations after relocation to an island-like environment with altered dietary resources

We observed a reaction to “colonisation” of island-like habitats in morphology and life-history. Overall, those morphological and life-history traits occurred as a response to stress, however, they did not show clear directional changes (Fig. 7). Risk-taking behaviour remained the same across all generations and diets. Absence of directional changes may be explained by the insufficient time frame in our experiment. Evolutionary adaptation driven by natural selection requires more than three generations and depends on underlying genetic changes. Nevertheless, fast evolutionary changes are possible, as for example, shifts in the beak size in Galapagos finches caused by drought-induced changes in diet [24]. Another possible explanation is that the change in diet composition is not a strong driver of phenotypic adjustments, in contrast to the diet quality (i.e., energetic value). For example, mice fed on a high-quality diet showed reduced risk-taking, leading to a faster life-history within three generations [53].

In the founder generation (F0), changes in body mass, testis weight, and sperm count tend to respond to relocation to island-like environments. All F0 mice exhibit reduced body mass, and males increase reproductive investment, as they have more sperm and heavier testes relative to body weight. Altogether, this suggests that mice that experienced relocation have shifted their energy allocation toward reproduction and not growth following the stress of relocation. Such a response is consistent with life-history theory predicting an increase in reproduction under changed conditions or elevated risk [11, 46, 62]. However, our animals displayed no differences in survival compared with ML population, which contradicts classic life-history expectations, in which animals investing in reproduction have lower survival rates. Such trade-offs are usually caused by energetic constraints, which, owing to the *ad libitum* food in our enclosures, might have been relaxed. Additionally, it suggests that male reproductive investment (sperm quantity) is low-cost and does not strongly trade off with survival rate. The absence of differences in the female reproductive investment (age of first reproduction) suggests sex-specific allocation of energy [13]. Altogether, the F0 generation seems to prioritise reproduction to succeed in the population establishment.

In contrast, the F1 generation is strongly affected by carry-over effects from the parental (F0) generation, where offspring phenotypes reflect parental environments rather than the current environment. In variable environments, trade-offs between growth, reproduction and survival can lead to a generation-specific difference in those parameters [14]. For example, parents experiencing low dietary availability may produce offspring that grow differently or allocate energy differently [65]. In our study, F1 generation mice exhibited increased structural growth (vertebrae length) and delayed reproduction in females. Similarly to the parental generation, the F1 generation showed no differences in survival rate compared with ML population with exception for mice on Veggie diet. These differences from F0 generations reflect the different energy allocation in F1. Wild house mice have been shown to exhibit this effect, as the offspring of parents exposed to a food switch showed slower growth rates and different stress coping behaviour [44]. Another example of a tubeworm (*Galeolaria caespitosa*) exhibited a buffering effect: when male parents experienced environmental stress (an increase in water temperature), they “prepared” their offspring for these stressful conditions [27]. Thus, we can say that the F1 generation increased investment in growth and delayed reproduction, possibly constrained by parental stress.

In the following generations (F2 and F3), we do not see a clear direction of changes. We see that in the F2 generation, females on a Soft and Veggie diet also reproduce later, similar to the F1 generation. However, we do not see any differences in the growth rate or vertebral length compared to the F0 generation. The survival rate for F2 showed no differences from ML population for Hard and Soft diet animals, however was lower for the Veggie diet. In the F3 generation, significantly lower survival compared with the ML population was observed in all three treatments. The absence of a consistent direction of change suggests that the changes in previous generations were indeed driven by relocation stress and were plastic rather than beginning adaptations or the outcome of selection processes.

Interestingly the Veggie diet mice showed more pronounced differences than Hard and Soft diet mice across several traits such as reproduction and survival. As all diets were designed to provide similar caloric content, these differences are unlikely to be explained by energy intake, which is further supported by similar consumption rates per day (Table S3). Instead, they could be caused by diet composition. Diet quality, i.e., the net energy intake, alters reproductive and behavioural traits even more strongly during the first three generations [54]. Mice on higher quality diets consistently invest into faster and more reproduction while at the same time reducing risk-taking behaviour consistently [54].

To conclude, our results show that differences in diet composition alone cannot explain IS, instead, in order for IS to develop, perhaps we need differences in diet quality and/or additional environmental variation. Besides that, three generations are not enough for the development and expression of IS, and it needs a longer time frame. However, our results show that the first response to these island-like environments reflects an energy allocation trade-off and carry-over effects from the parental generation. Sudden environmental changes can initially trigger physiological responses that are associated with a stress response, before more specific adaptive adjustments occur [45]. The absence of behavioural changes across generations and diets suggests that the dietary manipulations used in the present study had a smaller influence on risk-taking behaviour than the manipulation of food quality and perceived predation risk used in previous studies [17, 43, 54]. The absence some of the expected results may also be due to the limitations of our experimental system. Each dietary regime in our study was assigned to a single population i.e. one enclosure each. While our semi-natural enclosures replicated several key aspects of island colonisation, such as isolation and novel diets, they did not replicate all the environmental pressures that are present on true islands. The absence of predators, interspecific competitors and other environmental challenges in particular may have reduced the strength of selection on behavioural and life-history traits. Nevertheless, our findings highlight the early stages of colonisation and invasion, and suggest that during the early stages, phenotypic plasticity and life-history shifts may play a greater role than behavioural changes. However, the mechanisms underlying these phenotypic responses cannot be determined from our experimental design. Specifically, we cannot distinguish between developmental plasticity, parental effects and phenotypic flexibility. Instead, our design allows us to determine whether populations exposed to different diets exhibit phenotypic responses during the early stages of population establishment.

## Conclusion

In conclusion, our findings suggest that, within three generations, differences in diet composition alone are insufficient to trigger the development of the morpho-physiological traits related to newly colonised, restricted island-like environments. Although changes in morphology and life-history characteristics were observed in the mice, these responses were neither directional nor consistent across generations. On the contrary, the observed patterns appear to reflect short-term stress responses, changes in energy allocation, and carry-over effects from the parental generation, rather than adaptive evolutionary changes. The founder generation prioritised reproductive investment, while the F1 generation exhibited enhanced growth but delayed reproduction, likely due to the carry-over effect from stress in the parental generation. Thus, our results suggest that the early stages of colonisation/ relocation are characterised primarily by phenotypic plasticity.

## Supplementary Information

Below is the link to the electronic supplementary material.

Supplementary Material 1

## Data Availability

The datasets used and/or analysed during the current study are available from the corresponding author on reasonable request.

## Abbreviations

IS: Island Syndrome
OF: Open Field
