# Supplementary material for: Behavioural, morphological and life-history adjustments in the early steps of colonising restricted semi-natural environments in wild house mice
Source: BMC Ecol Evol. 2026 Sep 24;26:83. doi: 10.1186/s12862-026-02590-0 (PMC13602551; doi:10.1186/s12862-026-02590-0)
Supplement: Supplementary file 1 [file 12862_2026_2590_MOESM1_ESM.docx]

**SUPPLEMENTARY MATERIAL**

**Behavioural, life-history and morphological adjustments in the early steps of colonising island-like habitats in wild house mice**

Ekaterina Gorshkova 1,2, Daniela E. Winkler 1,3, Anja Guenther 2,4, Christine Böhmer 1

1 - Zoology and Functional Morphology of Vertebrates, Zoological Institute, Christian-Albrechts-Universität zu Kiel, Am Botanischen Garten 3–9, 24118 Kiel, Germany

2 - RG Behavioural Ecology of individual differences, Max Planck Institute for Evolutionary Biology, 24306 Plön, Germany

3 - Bonn Institute for Organismic Biology (BIOB), Department Paleontology, University of Bonn, Nussallee 8, 51331 Bonn, Germany

4 - Zoologie und Tierökologie, Universität Hildesheim, Universitätsplatz 1, 31141 Hildesheim, Germany

**Figure S1**. Experimental timeline. The experiment began in autumn 2023 with the initial population being divided into a mainland control group (ML) and three experimental groups: Hard, Soft and Veggie. The timeline shows when each generation was terminated and the age range of the animals at the time of termination. The ML control population was terminated in spring 2024. Stars indicate the time points at which open field tests were conducted. At the time of behavioural testing, the animals were 3–5 months old.

**Table S1.** Sex ratio at the end of each generation by treatment

| **Diet** | **generation** | **m** | **f** | **total** | **male_percent** | **female_percent** | **sex_ratio** |
| --- | --- | --- | --- | --- | --- | --- | --- |
| **ML** | F6 | 13 | 18 | 31 | 41.94 | 58.06 | 13:18 |
| **Hard** | F0 | 8 | 16 | 24 | 33.33 | 66.67 | 8:16 |
| **Hard** | F1 | 27 | 51 | 78 | 34.62 | 65.38 | 27:51 |
| **Hard** | F2 | 23 | 18 | 41 | 56.10 | 43.90 | 23:18 |
| **Hard** | F3 | 25 | 47 | 72 | 34.72 | 65.28 | 25:47 |
| **Soft** | F0 | 9 | 18 | 27 | 33.33 | 66.67 | 9:18 |
| **Soft** | F1 | 20 | 42 | 62 | 32.26 | 67.74 | 20:24 |
| **Soft** | F2 | 14 | 26 | 40 | 35 | 65 | 14:26 |
| **Soft** | F3 | 33 | 34 | 67 | 49,25373134 | 50,74626866 | 33:34 |
| **Veggie** | F0 | 11 | 20 | 31 | 35,48387097 | 64,51612903 | 11:20 |
| **Veggie** | F1 | 21 | 35 | 56 | 37,5 | 62,5 | 21:35 |
| **Veggie** | F2 | 17 | 22 | 39 | 43,58974359 | 56,41025641 | 17:22 |
| **Veggie** | F3 | 41 | 43 | 84 | 48,80952381 | 51,19047619 | 41:43 |

**Table S2.** Number and and of animals used for dissection

| Diet | Generation | Males | Females | Age at dissection |
| --- | --- | --- | --- | --- |
| ML | F6 | 11 | 18 | 6-8 months |
| Hard | F0 | 8 | 16 | 7 months |
| Hard | F1 | 16 | 19 | 5-8 months |
| Hard | F2 | 12 | 17 | 5-9 months |
| Hard | F3 | 13 | 18 | 4-7 months |
| Soft | F0 | 9 | 18 | 7 months |
| Soft | F1 | 15 | 18 | 5-8 months |
| Soft | F2 | 13 | 18 | 5-9 months |
| Soft | F3 | 13 | 18 | 4-7 months |
| Veggie | F0 | 11 | 20 | 7 months |
| Veggie | F1 | 15 | 19 | 5-8 months |
| Veggie | F2 | 11 | 19 | 5-9 months |
| Veggie | F3 | 13 | 18 | 4-7 months |

**Table S3**. Detailed diet composition

| Diet | Base diet composition | Supplement (% of final diet) | Supplement ingredients |
| --- | --- | --- | --- |
| Hard | 25% pea grits, 22% wheat, 22% barley, 21% oats, 10% black sunflower seeds | RuCat Premium 3-Mix (6.6%) | Cereals, meat and animal by-products, vegetable protein extracts, oils and fats, minerals |
| Soft | 25% pea flakes, 22% wheat flakes, 22% barley flakes, 21% oat flakes, 10% hulled sunflower kernels | *Hermetia* larvae (5.0%) |  |
| Veggie | 25% pea flakes, 22% wheat flakes, 22% barley flakes, 21% oat flakes, 10% hulled sunflower kernels | Vegetable mix for dogs (BARF mixture)  (12.5%) | Pea flakes, lucerne cubes, carrot flakes, potato cubes, parsnip cubes, beetroot cubes |

**Table S4.** Pairwise comparisons of consumption over 24 a hour period. Consumption was analysed using a one-way ANOVA with diet as the explanatory variable, followed by Tukey’s HSD pairwise comparisons. Consumption was measured per cage, with two females approximately 5 months old housed together and sharing one food plate. Sample sizes represent cage-level observations: Hard: n = 6 cages (12 mice), Soft: n = 7 cages (14 mice), and Veggie: n = 6 cages (12 mice).

| aov(consumed ~ diet, data= consumption) | | | | |
| --- | --- | --- | --- | --- |
| Comparison | diff | lwr | upr | p.adj |
| Soft-Hard | -3.92 | -9.92 | 2.08 | 0.24 |
| Veggie-Hard | -0.94 | -7.17 | 5.28 | 0.92 |
| Veggie-Soft | 2.97 | -3.03 | 8.97 | 0.43 |

**Figure S2**. Lateral X-ray of a wild house mouse illustrating centrum length measurement of the C2, L1, Ca1.

**Table S5**. Linear model estimates for the Open Field test in comparison to control mice (F6 ML).

| **Distance covered (cm)**  **model <- lm(Distance_Total_cm ~ treatment, data = data)** | | |  |
| --- | --- | --- | --- |
| *Predictors* | *Estimates* | *CI* | *p* |
| (Intercept) | 2531.37 | 2199.10 – 2863.65 | **<0.001** |
| group [F0_Hard] | 177.61 | -287.76 – 642.97 | 0.453 |
| group [F1_Hard] | -428.50 | -898.41 – 41.41 | 0.074 |
| group [F3_Hard] | -93.24 | -563.14 – 376.67 | 0.696 |
| group [F0_Soft] | 146.44 | -323.46 – 616.35 | 0.540 |
| group [F1_Soft] | 36.20 | -433.71 – 506.10 | 0.880 |
| group [F3_Soft] | -405.19 | -875.10 – 64.71 | 0.091 |
| group [F0_Veggie] | 447.49 | -22.41 – 917.40 | 0.062 |
| group [F1_Veggie] | 212.72 | -252.65 – 678.08 | 0.369 |
| group [F3_Veggie] | 47.47 | -422.43 – 517.38 | 0.842 |
| **Time in centre (s)**  **model <- lm(time_centre_s ~ treatment, data = data)** | | |  |
| *Predictors* | *Estimates* | *CI* | *p* |
| (Intercept) | 14.53 | 8.53 – 20.53 | **<0.001** |
| group [F0_Hard] | 1.58 | -6.82 – 9.99 | 0.711 |
| group [F1_Hard] | 6.63 | -1.85 – 15.12 | 0.125 |
| group [F3_Hard] | -4.78 | -13.26 – 3.71 | 0.269 |
| group [F0_Soft] | -4.75 | -13.24 – 3.74 | 0.271 |
| group [F1_Soft] | -2.24 | -10.73 – 6.25 | 0.604 |
| group [F3_Soft] | -8.77 | -17.26 – -0.29 | **0.043** |
| group [F0_Veggie] | -6.66 | -15.14 – 1.83 | 0.124 |
| group [F1_Veggie] | -2.06 | -10.46 – 6.35 | 0.630 |
| group [F3_Veggie] | -5.30 | -13.78 – 3.19 | 0.220 |
| **Mobility %**  **model <- lm(Mobility_Duration_percent ~ treatment, data = data)** | | |  |
| *Predictors* | *Estimates* | *CI* | *p* |
| (Intercept) | 90.81 | 88.71 – 92.91 | **<0.001** |
| group [F0_Hard] | -2.29 | -5.23 – 0.65 | 0.127 |
| group [F1_Hard] | 2.68 | -0.29 – 5.65 | 0.077 |
| group [F3_Hard] | 0.61 | -2.36 – 3.58 | 0.685 |
| group [F0_Soft] | -2.13 | -5.10 – 0.84 | 0.158 |
| group [F1_Soft] | 0.32 | -2.65 – 3.29 | 0.832 |
| group [F3_Soft] | 0.81 | -2.16 – 3.78 | 0.592 |
| group [F0_Veggie] | -3.27 | -6.24 – -0.30 | **0.031** |
| group [F1_Veggie] | -1.70 | -4.64 – 1.24 | 0.256 |
| group [F3_Veggie] | -2.65 | -5.62 – 0.32 | 0.080 |
| **Temperature difference** | | |  |
| *Predictors* | *Estimates* | *CI* | *p* |
| (Intercept) | 0.47 | 0.17 – 0.77 | **0.003** |
| group [F0_Hard] | 0.39 | -0.03 – 0.82 | 0.070 |
| group [F1_Hard] | -0.04 | -0.47 – 0.39 | 0.838 |
| group [F3_Hard] | -0.18 | -0.61 – 0.25 | 0.412 |
| group [F0_Soft] | -0.00 | -0.43 – 0.43 | 0.998 |
| group [F1_Soft] | 0.03 | -0.40 – 0.46 | 0.886 |
| group [F3_Soft] | -0.24 | -0.67 – 0.19 | 0.266 |
| group [F0_Veggie] | -0.32 | -0.75 – 0.11 | 0.140 |
| group [F1_Veggie] | -0.08 | -0.51 – 0.35 | 0.713 |
| group [F3_Veggie] | 0.34 | -0.09 – 0.77 | 0.116 |

**Table S6.** Linear model estimates for the Analysis of vertebrae counts

| **Vertebral column count up to the sacrum** | | |  |
| --- | --- | --- | --- |
| *Predictors* | *Estimates* | *CI* | *p* |
| (Intercept) | 29.91 | 29.79 – 30.03 | **<0.001** |
| generation [F0] | -0.12 | -0.26 – 0.02 | 0.106 |
| generation [F1] | 0.10 | -0.04 – 0.23 | 0.176 |
| generation [F2] | 0.06 | -0.08 – 0.20 | 0.408 |
| generation [F3] | 0.01 | -0.13 – 0.15 | 0.899 |
| treatment [Hard] | -0.02 | -0.10 – 0.06 | 0.599 |
| treatment [Soft] | 0.01 | -0.07 – 0.09 | 0.819 |
| sex [m] | -0.05 | -0.12 – 0.01 | 0.102 |
| Broken Tail [1] | 0.02 | -0.06 – 0.10 | 0.607 |
| **Vertebral count in caudal region** | | |  |
| *Predictors* | *Estimates* | *CI* | *p* |
| (Intercept) | 30.22 | 29.44 – 30.99 | **<0.001** |
| generation [F0] | -1.07 | -2.00 – -0.14 | **0.024** |
| generation [F1] | 0.64 | -0.27 – 1.54 | 0.167 |
| generation [F2] | -0.14 | -1.05 – 0.77 | 0.761 |
| generation [F3] | -0.38 | -1.29 – 0.53 | 0.413 |
| treatment [Hard] | -0.13 | -0.64 – 0.38 | 0.613 |
| treatment [Soft] | 0.04 | -0.47 – 0.55 | 0.871 |
| sex [m] | -0.30 | -0.72 – 0.13 | 0.173 |
| Broken Tail [1] | -4.20 | -4.71 – -3.68 | **<0.001** |

**Table S7.** Post-hoc Tukey comparisons for C2, L1, and Ca1the second cervical (C2), first lumbar (L1), and first caudal (Ca1) vertebrae

| C2  model <- lm(C2_length ~ group + sex + body_mass, data = xray2_c2)  emmeans(model, pairwise ~ group, adjust = "tukey") | | | | | |
| --- | --- | --- | --- | --- | --- |
| contrast | stimate | SE | df | t.ratio | p.value |
| F6_ML - F0_Hard | -0.18091 | 0.0654 | 357 | -2.766 | 0.2230 |
| F6_ML - F1_Hard | -0.61699 | 0.0600 | 357 | -10.275 | <.0001 |
| F6_ML - F2_Hard | -0.04528 | 0.0620 | 357 | -0.731 | 0.9999 |
| F6_ML - F3_Hard | -0.03741 | 0.0614 | 357 | -0.609 | 1.0000 |
| F6_ML - F0_Soft | -0.01065 | 0.0643 | 357 | -0.166 | 1.0000 |
| F6_ML - F1_Soft | -0.66236 | 0.0644 | 357 | -10.284 | <.0001 |
| F6_ML - F2_Soft | 0.00525 | 0.0617 | 357 | 0.085 | 1.0000 |
| F6_ML - F3_Soft | -0.04701 | 0.0614 | 357 | -0.765 | 0.9999 |
| F6_ML - F0_Veggie | -0.05542 | 0.0610 | 357 | -0.908 | 0.9995 |
| F6_ML - F1_Veggie | -0.71103 | 0.0650 | 357 | -10.935 | <.0001 |
| F6_ML - F2_Veggie | -0.06780 | 0.0613 | 357 | -1.106 | 0.9966 |
| F6_ML - F3_Veggie | -0.01385 | 0.0608 | 357 | -0.228 | 1.0000 |
| F0_Hard - F1_Hard | -0.43608 | 0.0630 | 357 | -6.927 | <.0001 |
| F0_Hard - F2_Hard | 0.13562 | 0.0651 | 357 | 2.082 | 0.6764 |
| F0_Hard - F3_Hard | 0.14349 | 0.0635 | 357 | 2.261 | 0.5470 |
| F0_Hard - F0_Soft | 0.17025 | 0.0654 | 357 | 2.602 | 0.3129 |
| F0_Hard - F1_Soft | -0.48145 | 0.0675 | 357 | -7.131 | <.0001 |
| F0_Hard - F2_Soft | 0.18615 | 0.0657 | 357 | 2.835 | 0.1911 |
| F0_Hard - F3_Soft | 0.13389 | 0.0629 | 357 | 2.130 | 0.6425 |
| F0_Hard - F0_Veggie | 0.12549 | 0.0629 | 357 | 1.994 | 0.7362 |
| F0_Hard - F1_Veggie | -0.53013 | 0.0671 | 357 | -7.896 | <.0001 |
| F0_Hard - F2_Veggie | 0.11310 | 0.0638 | 357 | 1.772 | 0.8624 |
| F0_Hard - F3_Veggie | 0.16706 | 0.0634 | 357 | 2.636 | 0.2928 |
| F1_Hard - F2_Hard | 0.57171 | 0.0589 | 357 | 9.707 | <.0001 |
| F1_Hard - F3_Hard | 0.57957 | 0.0586 | 357 | 9.888 | <.0001 |
| F1_Hard - F0_Soft | 0.60633 | 0.0619 | 357 | 9.789 | <.0001 |
| F1_Hard - F1_Soft | -0.04537 | 0.0613 | 357 | -0.740 | 0.9999 |
| F1_Hard - F2_Soft | 0.62223 | 0.0585 | 357 | 10.645 | <.0001 |
| F1_Hard - F3_Soft | 0.56998 | 0.0587 | 357 | 9.702 | <.0001 |
| F1_Hard - F0_Veggie | 0.56157 | 0.0583 | 357 | 9.638 | <.0001 |
| F1_Hard - F1_Veggie | -0.09404 | 0.0622 | 357 | -1.512 | 0.9526 |
| F1_Hard - F2_Veggie | 0.54918 | 0.0584 | 357 | 9.405 | <.0001 |
| F1_Hard - F3_Veggie | 0.60314 | 0.0579 | 357 | 10.425 | <.0001 |
| F2_Hard - F3_Hard | 0.00787 | 0.0608 | 357 | 0.129 | 1.0000 |
| F2_Hard - F0_Soft | 0.03463 | 0.0642 | 357 | 0.539 | 1.0000 |
| F2_Hard - F1_Soft | -0.61708 | 0.0631 | 357 | -9.778 | <.0001 |
| F2_Hard - F2_Soft | 0.05053 | 0.0600 | 357 | 0.842 | 0.9998 |
| F2_Hard - F3_Soft | -0.00173 | 0.0611 | 357 | -0.028 | 1.0000 |
| F2_Hard - F0_Veggie | -0.01014 | 0.0605 | 357 | -0.168 | 1.0000 |
| F2_Hard - F1_Veggie | -0.66575 | 0.0643 | 357 | -10.359 | <.0001 |
| F2_Hard - F2_Veggie | -0.02252 | 0.0604 | 357 | -0.373 | 1.0000 |
| F2_Hard - F3_Veggie | 0.03143 | 0.0599 | 357 | 0.525 | 1.0000 |
| F3_Hard - F0_Soft | 0.02676 | 0.0623 | 357 | 0.430 | 1.0000 |
| F3_Hard - F1_Soft | -0.62494 | 0.0633 | 357 | -9.880 | <.0001 |
| F3_Hard - F2_Soft | 0.04266 | 0.0609 | 357 | 0.700 | 1.0000 |
| F3_Hard - F3_Soft | -0.00960 | 0.0594 | 357 | -0.162 | 1.0000 |
| F3_Hard - F0_Veggie | -0.01800 | 0.0592 | 357 | -0.304 | 1.0000 |
| F3_Hard - F1_Veggie | -0.67362 | 0.0634 | 357 | -10.628 | <.0001 |
| F3_Hard - F2_Veggie | -0.03039 | 0.0598 | 357 | -0.508 | 1.0000 |
| F3_Hard - F3_Veggie | 0.02357 | 0.0593 | 357 | 0.398 | 1.0000 |
| F0_Soft - F1_Soft | -0.65170 | 0.0666 | 357 | -9.779 | <.0001 |
| F0_Soft - F2_Soft | 0.01590 | 0.0649 | 357 | 0.245 | 1.0000 |
| F0_Soft - F3_Soft | -0.03636 | 0.0615 | 357 | -0.591 | 1.0000 |
| F0_Soft - F0_Veggie | -0.04476 | 0.0617 | 357 | -0.726 | 1.0000 |
| F0_Soft - F1_Veggie | -0.70038 | 0.0661 | 357 | -10.601 | <.0001 |
| F0_Soft - F2_Veggie | -0.05715 | 0.0627 | 357 | -0.911 | 0.9995 |
| F0_Soft - F3_Veggie | -0.00319 | 0.0623 | 357 | -0.051 | 1.0000 |
| F1_Soft - F2_Soft | 0.66760 | 0.0624 | 357 | 10.696 | <.0001 |
| F1_Soft - F3_Soft | 0.61535 | 0.0636 | 357 | 9.679 | <.0001 |
| F1_Soft - F0_Veggie | 0.60694 | 0.0630 | 357 | 9.637 | <.0001 |
| F1_Soft - F1_Veggie | -0.04867 | 0.0665 | 357 | -0.732 | 0.9999 |
| F1_Soft - F2_Veggie | 0.59455 | 0.0629 | 357 | 9.456 | <.0001 |
| F1_Soft - F3_Veggie | 0.64851 | 0.0624 | 357 | 10.397 | <.0001 |
| F2_Soft - F3_Soft | -0.05226 | 0.0617 | 357 | -0.847 | 0.9998 |
| F2_Soft - F0_Veggie | -0.06066 | 0.0607 | 357 | -1.000 | 0.9987 |
| F2_Soft - F1_Veggie | -0.71628 | 0.0643 | 357 | -11.141 | <.0001 |
| F2_Soft - F2_Veggie | -0.07305 | 0.0601 | 357 | -1.215 | 0.9921 |
| F2_Soft - F3_Veggie | -0.01909 | 0.0597 | 357 | -0.320 | 1.0000 |
| F3_Soft - F0_Veggie | -0.00841 | 0.0588 | 357 | -0.143 | 1.0000 |
| F3_Soft - F1_Veggie | -0.66402 | 0.0632 | 357 | -10.514 | <.0001 |
| F3_Soft - F2_Veggie | -0.02079 | 0.0598 | 357 | -0.348 | 1.0000 |
| F3_Soft - F3_Veggie | 0.03317 | 0.0593 | 357 | 0.560 | 1.0000 |
| F0_Veggie - F1_Veggie | -0.65561 | 0.0630 | 357 | -10.403 | <.0001 |
| F0_Veggie - F2_Veggie | -0.01239 | 0.0594 | 357 | -0.209 | 1.0000 |
| F0_Veggie - F3_Veggie | 0.04157 | 0.0589 | 357 | 0.706 | 1.0000 |
| F1_Veggie - F2_Veggie | 0.64323 | 0.0635 | 357 | 10.133 | <.0001 |
| F1_Veggie - F3_Veggie | 0.69719 | 0.0629 | 357 | 11.077 | <.0001 |
| F2_Veggie - F3_Veggie | 0.05396 | 0.0592 | 357 | 0.912 | 0.9995 |
| L1  model <- lm(L1_length ~ group + sex + body_mass, data = xray2)  emmeans(model, pairwise ~ group, adjust = "tukey") | | | | | |
| contrast | estimate | SE | df | t.ratio | p.value |
| F6_ML - F0_Hard | 0.15807 | 0.0539 | 379 | 2.933 | 0.1509 |
| F6_ML - F1_Hard | -0.66597 | 0.0487 | 379 | -13.666 | <.0001 |
| F6_ML - F2_Hard | 0.13421 | 0.0510 | 379 | 2.634 | 0.2938 |
| F6_ML - F3_Hard | 0.16171 | 0.0506 | 379 | 3.198 | 0.0746 |
| F6_ML - F0_Soft | 0.12459 | 0.0525 | 379 | 2.371 | 0.4666 |
| F6_ML - F1_Soft | -0.69769 | 0.0496 | 379 | -14.077 | <.0001 |
| F6_ML - F2_Soft | 0.14985 | 0.0507 | 379 | 2.958 | 0.1419 |
| F6_ML - F3_Soft | 0.30377 | 0.0506 | 379 | 6.007 | <.0001 |
| F6_ML - F0_Veggie | 0.17856 | 0.0502 | 379 | 3.555 | 0.0248 |
| F6_ML - F1_Veggie | -0.77909 | 0.0490 | 379 | -15.902 | <.0001 |
| F6_ML - F2_Veggie | 0.10054 | 0.0504 | 379 | 1.993 | 0.7369 |
| F6_ML - F3_Veggie | 0.27375 | 0.0500 | 379 | 5.470 | <.0001 |
| F0_Hard - F1_Hard | -0.82405 | 0.0518 | 379 | -15.923 | <.0001 |
| F0_Hard - F2_Hard | -0.02386 | 0.0540 | 379 | -0.442 | 1.0000 |
| F0_Hard - F3_Hard | 0.00363 | 0.0527 | 379 | 0.069 | 1.0000 |
| F0_Hard - F0_Soft | -0.03349 | 0.0539 | 379 | -0.622 | 1.0000 |
| F0_Hard - F1_Soft | -0.85576 | 0.0529 | 379 | -16.166 | <.0001 |
| F0_Hard - F2_Soft | -0.00823 | 0.0544 | 379 | -0.151 | 1.0000 |
| F0_Hard - F3_Soft | 0.14570 | 0.0522 | 379 | 2.790 | 0.2110 |
| F0_Hard - F0_Veggie | 0.02049 | 0.0523 | 379 | 0.392 | 1.0000 |
| F0_Hard - F1_Veggie | -0.93716 | 0.0517 | 379 | -18.140 | <.0001 |
| F0_Hard - F2_Veggie | -0.05754 | 0.0530 | 379 | -1.086 | 0.9972 |
| F0_Hard - F3_Veggie | 0.11568 | 0.0526 | 379 | 2.199 | 0.5928 |
| F1_Hard - F2_Hard | 0.80019 | 0.0482 | 379 | 16.588 | <.0001 |
| F1_Hard - F3_Hard | 0.82768 | 0.0481 | 379 | 17.211 | <.0001 |
| F1_Hard - F0_Soft | 0.79056 | 0.0504 | 379 | 15.688 | <.0001 |
| F1_Hard - F1_Soft | -0.03171 | 0.0467 | 379 | -0.679 | 1.0000 |
| F1_Hard - F2_Soft | 0.81582 | 0.0477 | 379 | 17.086 | <.0001 |
| F1_Hard - F3_Soft | 0.96975 | 0.0482 | 379 | 20.115 | <.0001 |
| F1_Hard - F0_Veggie | 0.84454 | 0.0478 | 379 | 17.669 | <.0001 |
| F1_Hard - F1_Veggie | -0.11312 | 0.0463 | 379 | -2.444 | 0.4153 |
| F1_Hard - F2_Veggie | 0.76651 | 0.0479 | 379 | 16.012 | <.0001 |
| F1_Hard - F3_Veggie | 0.93972 | 0.0474 | 379 | 19.815 | <.0001 |
| F2_Hard - F3_Hard | 0.02749 | 0.0504 | 379 | 0.545 | 1.0000 |
| F2_Hard - F0_Soft | -0.00963 | 0.0528 | 379 | -0.182 | 1.0000 |
| F2_Hard - F1_Soft | -0.83190 | 0.0489 | 379 | -17.017 | <.0001 |
| F2_Hard - F2_Soft | 0.01563 | 0.0498 | 379 | 0.314 | 1.0000 |
| F2_Hard - F3_Soft | 0.16956 | 0.0507 | 379 | 3.346 | 0.0483 |
| F2_Hard - F0_Veggie | 0.04435 | 0.0502 | 379 | 0.884 | 0.9996 |
| F2_Hard - F1_Veggie | -0.91330 | 0.0487 | 379 | -18.770 | <.0001 |
| F2_Hard - F2_Veggie | -0.03368 | 0.0501 | 379 | -0.672 | 1.0000 |
| F2_Hard - F3_Veggie | 0.13954 | 0.0497 | 379 | 2.805 | 0.2043 |
| F3_Hard - F0_Soft | -0.03712 | 0.0512 | 379 | -0.725 | 1.0000 |
| F3_Hard - F1_Soft | -0.85939 | 0.0492 | 379 | -17.483 | <.0001 |
| F3_Hard - F2_Soft | -0.01186 | 0.0505 | 379 | -0.235 | 1.0000 |
| F3_Hard - F3_Soft | 0.14207 | 0.0493 | 379 | 2.882 | 0.1707 |
| F3_Hard - F0_Veggie | 0.01686 | 0.0492 | 379 | 0.343 | 1.0000 |
| F3_Hard - F1_Veggie | -0.94080 | 0.0482 | 379 | -19.523 | <.0001 |
| F3_Hard - F2_Veggie | -0.06117 | 0.0497 | 379 | -1.232 | 0.9910 |
| F3_Hard - F3_Veggie | 0.11204 | 0.0492 | 379 | 2.275 | 0.5364 |
| F0_Soft - F1_Soft | -0.82227 | 0.0517 | 379 | -15.904 | <.0001 |
| F0_Soft - F2_Soft | 0.02526 | 0.0533 | 379 | 0.474 | 1.0000 |
| F0_Soft - F3_Soft | 0.17919 | 0.0506 | 379 | 3.543 | 0.0258 |
| F0_Soft - F0_Veggie | 0.05398 | 0.0507 | 379 | 1.064 | 0.9977 |
| F0_Soft - F1_Veggie | -0.90367 | 0.0502 | 379 | -17.991 | <.0001 |
| F0_Soft - F2_Veggie | -0.02405 | 0.0516 | 379 | -0.466 | 1.0000 |
| F0_Soft - F3_Veggie | 0.14916 | 0.0512 | 379 | 2.914 | 0.1583 |
| F1_Soft - F2_Soft | 0.84753 | 0.0481 | 379 | 17.616 | <.0001 |
| F1_Soft - F3_Soft | 1.00146 | 0.0495 | 379 | 20.231 | <.0001 |
| F1_Soft - F0_Veggie | 0.87625 | 0.0489 | 379 | 17.918 | <.0001 |
| F1_Soft - F1_Veggie | -0.08140 | 0.0472 | 379 | -1.725 | 0.8835 |
| F1_Soft - F2_Veggie | 0.79822 | 0.0487 | 379 | 16.375 | <.0001 |
| F1_Soft - F3_Veggie | 0.97143 | 0.0483 | 379 | 20.098 | <.0001 |
| F2_Soft - F3_Soft | 0.15393 | 0.0510 | 379 | 3.015 | 0.1226 |
| F2_Soft - F0_Veggie | 0.02872 | 0.0503 | 379 | 0.571 | 1.0000 |
| F2_Soft - F1_Veggie | -0.92893 | 0.0484 | 379 | -19.196 | <.0001 |
| F2_Soft - F2_Veggie | -0.04931 | 0.0499 | 379 | -0.988 | 0.9989 |
| F2_Soft - F3_Veggie | 0.12390 | 0.0495 | 379 | 2.502 | 0.3760 |
| F3_Soft - F0_Veggie | -0.12521 | 0.0489 | 379 | -2.563 | 0.3369 |
| F3_Soft - F1_Veggie | -1.08286 | 0.0481 | 379 | -22.491 | <.0001 |
| F3_Soft - F2_Veggie | -0.20324 | 0.0496 | 379 | -4.096 | 0.0035 |
| F3_Soft - F3_Veggie | -0.03002 | 0.0492 | 379 | -0.611 | 1.0000 |
| F0_Veggie - F1_Veggie | -0.95765 | 0.0478 | 379 | -20.014 | <.0001 |
| F0_Veggie - F2_Veggie | -0.07803 | 0.0493 | 379 | -1.583 | 0.9344 |
| F0_Veggie - F3_Veggie | 0.09519 | 0.0489 | 379 | 1.947 | 0.7665 |
| F1_Veggie - F2_Veggie | 0.87963 | 0.0481 | 379 | 18.287 | <.0001 |
| F1_Veggie - F3_Veggie | 1.05284 | 0.0477 | 379 | 22.088 | <.0001 |
| F2_Veggie - F3_Veggie | 0.17321 | 0.0492 | 379 | 3.523 | 0.0276 |
| Ca1  model <- lm(Ca1_length ~ group + sex + body_mass, data = xray2)  emmeans(model, pairwise ~ group, adjust = "tukey") | | | | | |
| contrast | estimate | SE | df | t.ratio | p.value |
| F6_ML - F0_Hard | 0.00680 | 0.0429 | 379 | 0.158 | 1.0000 |
| F6_ML - F1_Hard | -0.75863 | 0.0388 | 379 | -19.554 | <.0001 |
| F6_ML - F2_Hard | 0.05187 | 0.0406 | 379 | 1.279 | 0.9876 |
| F6_ML - F3_Hard | 0.06093 | 0.0403 | 379 | 1.514 | 0.9525 |
| F6_ML - F0_Soft | 0.06865 | 0.0418 | 379 | 1.641 | 0.9157 |
| F6_ML - F1_Soft | -0.74155 | 0.0395 | 379 | -18.793 | <.0001 |
| F6_ML - F2_Soft | 0.10927 | 0.0403 | 379 | 2.709 | 0.2518 |
| F6_ML - F3_Soft | 0.16074 | 0.0403 | 379 | 3.993 | 0.0052 |
| F6_ML - F0_Veggie | -0.03078 | 0.0400 | 379 | -0.770 | 0.9999 |
| F6_ML - F1_Veggie | -0.74680 | 0.0390 | 379 | -19.147 | <.0001 |
| F6_ML - F2_Veggie | 0.06194 | 0.0402 | 379 | 1.542 | 0.9454 |
| F6_ML - F3_Veggie | 0.16779 | 0.0398 | 379 | 4.211 | 0.0022 |
| F0_Hard - F1_Hard | -0.76543 | 0.0412 | 379 | -18.578 | <.0001 |
| F0_Hard - F2_Hard | 0.04508 | 0.0430 | 379 | 1.048 | 0.9980 |
| F0_Hard - F3_Hard | 0.05413 | 0.0420 | 379 | 1.290 | 0.9867 |
| F0_Hard - F0_Soft | 0.06185 | 0.0429 | 379 | 1.442 | 0.9670 |
| F0_Hard - F1_Soft | -0.74835 | 0.0421 | 379 | -17.757 | <.0001 |
| F0_Hard - F2_Soft | 0.10247 | 0.0433 | 379 | 2.368 | 0.4693 |
| F0_Hard - F3_Soft | 0.15394 | 0.0416 | 379 | 3.703 | 0.0150 |
| F0_Hard - F0_Veggie | -0.03758 | 0.0416 | 379 | -0.903 | 0.9995 |
| F0_Hard - F1_Veggie | -0.75360 | 0.0411 | 379 | -18.322 | <.0001 |
| F0_Hard - F2_Veggie | 0.05514 | 0.0422 | 379 | 1.307 | 0.9851 |
| F0_Hard - F3_Veggie | 0.16099 | 0.0419 | 379 | 3.843 | 0.0091 |
| F1_Hard - F2_Hard | 0.81051 | 0.0384 | 379 | 21.105 | <.0001 |
| F1_Hard - F3_Hard | 0.81956 | 0.0383 | 379 | 21.407 | <.0001 |
| F1_Hard - F0_Soft | 0.82728 | 0.0401 | 379 | 20.621 | <.0001 |
| F1_Hard - F1_Soft | 0.01709 | 0.0372 | 379 | 0.460 | 1.0000 |
| F1_Hard - F2_Soft | 0.86790 | 0.0380 | 379 | 22.832 | <.0001 |
| F1_Hard - F3_Soft | 0.91937 | 0.0384 | 379 | 23.954 | <.0001 |
| F1_Hard - F0_Veggie | 0.72785 | 0.0381 | 379 | 19.127 | <.0001 |
| F1_Hard - F1_Veggie | 0.01183 | 0.0368 | 379 | 0.321 | 1.0000 |
| F1_Hard - F2_Veggie | 0.82057 | 0.0381 | 379 | 21.531 | <.0001 |
| F1_Hard - F3_Veggie | 0.92642 | 0.0378 | 379 | 24.537 | <.0001 |
| F2_Hard - F3_Hard | 0.00906 | 0.0402 | 379 | 0.225 | 1.0000 |
| F2_Hard - F0_Soft | 0.01677 | 0.0420 | 379 | 0.399 | 1.0000 |
| F2_Hard - F1_Soft | -0.79342 | 0.0389 | 379 | -20.387 | <.0001 |
| F2_Hard - F2_Soft | 0.05740 | 0.0396 | 379 | 1.448 | 0.9660 |
| F2_Hard - F3_Soft | 0.10886 | 0.0403 | 379 | 2.698 | 0.2577 |
| F2_Hard - F0_Veggie | -0.08266 | 0.0399 | 379 | -2.070 | 0.6854 |
| F2_Hard - F1_Veggie | -0.79867 | 0.0387 | 379 | -20.617 | <.0001 |
| F2_Hard - F2_Veggie | 0.01007 | 0.0399 | 379 | 0.252 | 1.0000 |
| F2_Hard - F3_Veggie | 0.11591 | 0.0396 | 379 | 2.927 | 0.1533 |
| F3_Hard - F0_Soft | 0.00772 | 0.0408 | 379 | 0.189 | 1.0000 |
| F3_Hard - F1_Soft | -0.80248 | 0.0391 | 379 | -20.505 | <.0001 |
| F3_Hard - F2_Soft | 0.04834 | 0.0402 | 379 | 1.203 | 0.9928 |
| F3_Hard - F3_Soft | 0.09981 | 0.0392 | 379 | 2.543 | 0.3492 |
| F3_Hard - F0_Veggie | -0.09171 | 0.0391 | 379 | -2.343 | 0.4872 |
| F3_Hard - F1_Veggie | -0.80773 | 0.0384 | 379 | -21.055 | <.0001 |
| F3_Hard - F2_Veggie | 0.00101 | 0.0395 | 379 | 0.026 | 1.0000 |
| F3_Hard - F3_Veggie | 0.10686 | 0.0392 | 379 | 2.726 | 0.2431 |
| F0_Soft - F1_Soft | -0.81019 | 0.0412 | 379 | -19.683 | <.0001 |
| F0_Soft - F2_Soft | 0.04062 | 0.0424 | 379 | 0.958 | 0.9992 |
| F0_Soft - F3_Soft | 0.09209 | 0.0403 | 379 | 2.287 | 0.5280 |
| F0_Soft - F0_Veggie | -0.09943 | 0.0404 | 379 | -2.462 | 0.4032 |
| F0_Soft - F1_Veggie | -0.81545 | 0.0400 | 379 | -20.392 | <.0001 |
| F0_Soft - F2_Veggie | -0.00671 | 0.0411 | 379 | -0.163 | 1.0000 |
| F0_Soft - F3_Veggie | 0.09914 | 0.0408 | 379 | 2.432 | 0.4235 |
| F1_Soft - F2_Soft | 0.85082 | 0.0383 | 379 | 22.213 | <.0001 |
| F1_Soft - F3_Soft | 0.90228 | 0.0394 | 379 | 22.896 | <.0001 |
| F1_Soft - F0_Veggie | 0.71076 | 0.0389 | 379 | 18.256 | <.0001 |
| F1_Soft - F1_Veggie | -0.00525 | 0.0376 | 379 | -0.140 | 1.0000 |
| F1_Soft - F2_Veggie | 0.80349 | 0.0388 | 379 | 20.705 | <.0001 |
| F1_Soft - F3_Veggie | 0.90933 | 0.0385 | 379 | 23.631 | <.0001 |
| F2_Soft - F3_Soft | 0.05146 | 0.0406 | 379 | 1.266 | 0.9886 |
| F2_Soft - F0_Veggie | -0.14005 | 0.0400 | 379 | -3.500 | 0.0297 |
| F2_Soft - F1_Veggie | -0.85607 | 0.0385 | 379 | -22.221 | <.0001 |
| F2_Soft - F2_Veggie | -0.04733 | 0.0397 | 379 | -1.192 | 0.9933 |
| F2_Soft - F3_Veggie | 0.05851 | 0.0394 | 379 | 1.484 | 0.9589 |
| F3_Soft - F0_Veggie | -0.19152 | 0.0389 | 379 | -4.924 | 0.0001 |
| F3_Soft - F1_Veggie | -0.90754 | 0.0383 | 379 | -23.677 | <.0001 |
| F3_Soft - F2_Veggie | -0.09880 | 0.0395 | 379 | -2.501 | 0.3769 |
| F3_Soft - F3_Veggie | 0.00705 | 0.0392 | 379 | 0.180 | 1.0000 |
| F0_Veggie - F1_Veggie | -0.71602 | 0.0381 | 379 | -18.796 | <.0001 |
| F0_Veggie - F2_Veggie | 0.09272 | 0.0392 | 379 | 2.362 | 0.4730 |
| F0_Veggie - F3_Veggie | 0.19857 | 0.0389 | 379 | 5.101 | <.0001 |
| F1_Veggie - F2_Veggie | 0.80874 | 0.0383 | 379 | 21.119 | <.0001 |
| F1_Veggie - F3_Veggie | 0.91459 | 0.0379 | 379 | 24.101 | <.0001 |
| F2_Veggie - F3_Veggie | 0.10585 | 0.0391 | 379 | 2.704 | 0.2546 |

**Table S8.** Post-hoc Tukey comparisons for sperm count and testie mass. Estimates and SE are expressed as mg testis mass per g body mass.

| sperm  model <- lm(sperm_weight ~ group, data = sperm)  emmeans(model, pairwise ~ group, adjust = "tukey") | | | | | |
| --- | --- | --- | --- | --- | --- |
| contrast | estimate | SE | df | t.ratio | p.value |
| F6_ML - F0_Hard | -298743.6 | 54065.34 | 147 | -5.526 | <.0001 |
| F6_ML - F1_Hard | 26503.6 | 45573.15 | 147 | 0.582 | 1.0000 |
| F6_ML - F2_Hard | 45344.4 | 48569.14 | 147 | 0.934 | 0.9993 |
| F6_ML - F3_Hard | -10317.0 | 47667.36 | 147 | -0.216 | 1.0000 |
| F6_ML - F0_Soft | -246626.3 | 52297.49 | 147 | -4.716 | 0.0004 |
| F6_ML - F1_Soft | -86277.5 | 46187.90 | 147 | -1.868 | 0.8109 |
| F6_ML - F2_Soft | 4673.9 | 47667.36 | 147 | 0.098 | 1.0000 |
| F6_ML - F3_Soft | 2066.6 | 47667.36 | 147 | 0.043 | 1.0000 |
| F6_ML - F0_Veggie | -130794.6 | 49613.76 | 147 | -2.636 | 0.2987 |
| F6_ML - F1_Veggie | 25365.6 | 46187.90 | 147 | 0.549 | 1.0000 |
| F6_ML - F2_Veggie | 161461.2 | 49613.76 | 147 | 3.254 | 0.0691 |
| F6_ML - F3_Veggie | -6248.7 | 47667.36 | 147 | -0.131 | 1.0000 |
| F0_Hard - F1_Hard | 325247.1 | 50383.01 | 147 | 6.455 | <.0001 |
| F0_Hard - F2_Hard | 344087.9 | 53108.36 | 147 | 6.479 | <.0001 |
| F0_Hard - F3_Hard | 288426.5 | 52284.92 | 147 | 5.516 | <.0001 |
| F0_Hard - F0_Soft | 52117.2 | 56538.16 | 147 | 0.922 | 0.9994 |
| F0_Hard - F1_Soft | 212466.0 | 50939.75 | 147 | 4.171 | 0.0034 |
| F0_Hard - F2_Soft | 303417.4 | 52284.92 | 147 | 5.803 | <.0001 |
| F0_Hard - F3_Soft | 300810.2 | 52284.92 | 147 | 5.753 | <.0001 |
| F0_Hard - F0_Veggie | 167949.0 | 54065.34 | 147 | 3.106 | 0.1029 |
| F0_Hard - F1_Veggie | 324109.2 | 50939.75 | 147 | 6.363 | <.0001 |
| F0_Hard - F2_Veggie | 460204.7 | 54065.34 | 147 | 8.512 | <.0001 |
| F0_Hard - F3_Veggie | 292494.8 | 52284.92 | 147 | 5.594 | <.0001 |
| F1_Hard - F2_Hard | 18840.8 | 44433.64 | 147 | 0.424 | 1.0000 |
| F1_Hard - F3_Hard | -36820.6 | 43446.10 | 147 | -0.848 | 0.9997 |
| F1_Hard - F0_Soft | -273129.9 | 48481.07 | 147 | -5.634 | <.0001 |
| F1_Hard - F1_Soft | -112781.1 | 41817.56 | 147 | -2.697 | 0.2651 |
| F1_Hard - F2_Soft | -21829.7 | 43446.10 | 147 | -0.502 | 1.0000 |
| F1_Hard - F3_Soft | -24437.0 | 43446.10 | 147 | -0.562 | 1.0000 |
| F1_Hard - F0_Veggie | -157298.1 | 45573.15 | 147 | -3.452 | 0.0390 |
| F1_Hard - F1_Veggie | -1137.9 | 41817.56 | 147 | -0.027 | 1.0000 |
| F1_Hard - F2_Veggie | 134957.6 | 45573.15 | 147 | 2.961 | 0.1478 |
| F1_Hard - F3_Veggie | -32752.3 | 43446.10 | 147 | -0.754 | 0.9999 |
| F2_Hard - F3_Hard | -55661.4 | 46579.11 | 147 | -1.195 | 0.9928 |
| F2_Hard - F0_Soft | -291970.7 | 51307.55 | 147 | -5.691 | <.0001 |
| F2_Hard - F1_Soft | -131621.9 | 45063.93 | 147 | -2.921 | 0.1627 |
| F2_Hard - F2_Soft | -40670.5 | 46579.11 | 147 | -0.873 | 0.9996 |
| F2_Hard - F3_Soft | -43277.8 | 46579.11 | 147 | -0.929 | 0.9993 |
| F2_Hard - F0_Veggie | -176139.0 | 48569.14 | 147 | -3.627 | 0.0226 |
| F2_Hard - F1_Veggie | -19978.7 | 45063.93 | 147 | -0.443 | 1.0000 |
| F2_Hard - F2_Veggie | 116116.8 | 48569.14 | 147 | 2.391 | 0.4564 |
| F2_Hard - F3_Veggie | -51593.1 | 46579.11 | 147 | -1.108 | 0.9964 |
| F3_Hard - F0_Soft | -236309.3 | 50454.73 | 147 | -4.684 | 0.0005 |
| F3_Hard - F1_Soft | -75960.5 | 44090.52 | 147 | -1.723 | 0.8822 |
| F3_Hard - F2_Soft | 14990.9 | 45638.02 | 147 | 0.328 | 1.0000 |
| F3_Hard - F3_Soft | 12383.6 | 45638.02 | 147 | 0.271 | 1.0000 |
| F3_Hard - F0_Veggie | -120477.6 | 47667.36 | 147 | -2.527 | 0.3647 |
| F3_Hard - F1_Veggie | 35682.6 | 44090.52 | 147 | 0.809 | 0.9998 |
| F3_Hard - F2_Veggie | 171778.2 | 47667.36 | 147 | 3.604 | 0.0244 |
| F3_Hard - F3_Veggie | 4068.3 | 45638.02 | 147 | 0.089 | 1.0000 |
| F0_Soft - F1_Soft | 160348.8 | 49059.40 | 147 | 3.268 | 0.0665 |
| F0_Soft - F2_Soft | 251300.2 | 50454.73 | 147 | 4.981 | 0.0001 |
| F0_Soft - F3_Soft | 248692.9 | 50454.73 | 147 | 4.929 | 0.0002 |
| F0_Soft - F0_Veggie | 115831.7 | 52297.49 | 147 | 2.215 | 0.5821 |
| F0_Soft - F1_Veggie | 271991.9 | 49059.40 | 147 | 5.544 | <.0001 |
| F0_Soft - F2_Veggie | 408087.5 | 52297.49 | 147 | 7.803 | <.0001 |
| F0_Soft - F3_Veggie | 240377.6 | 50454.73 | 147 | 4.764 | 0.0003 |
| F1_Soft - F2_Soft | 90951.4 | 44090.52 | 147 | 2.063 | 0.6894 |
| F1_Soft - F3_Soft | 88344.1 | 44090.52 | 147 | 2.004 | 0.7288 |
| F1_Soft - F0_Veggie | -44517.0 | 46187.90 | 147 | -0.964 | 0.9990 |
| F1_Soft - F1_Veggie | 111643.2 | 42486.69 | 147 | 2.628 | 0.3036 |
| F1_Soft - F2_Veggie | 247738.7 | 46187.90 | 147 | 5.364 | <.0001 |
| F1_Soft - F3_Veggie | 80028.8 | 44090.52 | 147 | 1.815 | 0.8391 |
| F2_Soft - F3_Soft | -2607.2 | 45638.02 | 147 | -0.057 | 1.0000 |
| F2_Soft - F0_Veggie | -135468.4 | 47667.36 | 147 | -2.842 | 0.1949 |
| F2_Soft - F1_Veggie | 20691.8 | 44090.52 | 147 | 0.469 | 1.0000 |
| F2_Soft - F2_Veggie | 156787.3 | 47667.36 | 147 | 3.289 | 0.0627 |
| F2_Soft - F3_Veggie | -10922.6 | 45638.02 | 147 | -0.239 | 1.0000 |
| F3_Soft - F0_Veggie | -132861.2 | 47667.36 | 147 | -2.787 | 0.2196 |
| F3_Soft - F1_Veggie | 23299.0 | 44090.52 | 147 | 0.528 | 1.0000 |
| F3_Soft - F2_Veggie | 159394.6 | 47667.36 | 147 | 3.344 | 0.0536 |
| F3_Soft - F3_Veggie | -8315.3 | 45638.02 | 147 | -0.182 | 1.0000 |
| F0_Veggie - F1_Veggie | 156160.2 | 46187.90 | 147 | 3.381 | 0.0482 |
| F0_Veggie - F2_Veggie | 292255.8 | 49613.76 | 147 | 5.891 | <.0001 |
| F0_Veggie - F3_Veggie | 124545.9 | 47667.36 | 147 | 2.613 | 0.3123 |
| F1_Veggie - F2_Veggie | 136095.6 | 46187.90 | 147 | 2.947 | 0.1531 |
| F1_Veggie - F3_Veggie | -31614.3 | 44090.52 | 147 | -0.717 | 1.0000 |
| F2_Veggie - F3_Veggie | -167709.9 | 47667.36 | 147 | -3.518 | 0.0318 |
| testis  model <- lm(testis_index ~ group, data = sperm)  emmeans(model, pairwise ~ group, adjust = "tukey") | | | | | |
| contrast | estimate | SE | df | t.ratio | p.value |
| F6_ML - F0_Hard | -2.226397 | 0.5843639 | 147 | -3.810 | 0.0124 |
| F6_ML - F1_Hard | -0.090546 | 0.4925763 | 147 | -0.184 | 1.0000 |
| F6_ML - F2_Hard | -1.150238 | 0.5249584 | 147 | -2.191 | 0.5992 |
| F6_ML - F3_Hard | -0.682759 | 0.5152115 | 147 | -1.325 | 0.9825 |
| F6_ML - F0_Soft | -3.261613 | 0.5652562 | 147 | -5.770 | <.0001 |
| F6_ML - F1_Soft | -0.398610 | 0.4992208 | 147 | -0.798 | 0.9999 |
| F6_ML - F2_Soft | 0.166446 | 0.5152115 | 147 | 0.323 | 1.0000 |
| F6_ML - F3_Soft | 0.268270 | 0.5152115 | 147 | 0.521 | 1.0000 |
| F6_ML - F0_Veggie | -0.887241 | 0.5362491 | 147 | -1.655 | 0.9090 |
| F6_ML - F1_Veggie | -0.558044 | 0.4992208 | 147 | -1.118 | 0.9960 |
| F6_ML - F2_Veggie | 0.492820 | 0.5362491 | 147 | 0.919 | 0.9994 |
| F6_ML - F3_Veggie | -0.083556 | 0.5152115 | 147 | -0.162 | 1.0000 |
| F0_Hard - F1_Hard | 2.135851 | 0.5445636 | 147 | 3.922 | 0.0084 |
| F0_Hard - F2_Hard | 1.076159 | 0.5740204 | 147 | 1.875 | 0.8071 |
| F0_Hard - F3_Hard | 1.543638 | 0.5651203 | 147 | 2.732 | 0.2470 |
| F0_Hard - F0_Soft | -1.035217 | 0.6110914 | 147 | -1.694 | 0.8940 |
| F0_Hard - F1_Soft | 1.827787 | 0.5505810 | 147 | 3.320 | 0.0575 |
| F0_Hard - F2_Soft | 2.392843 | 0.5651203 | 147 | 4.234 | 0.0027 |
| F0_Hard - F3_Soft | 2.494667 | 0.5651203 | 147 | 4.414 | 0.0013 |
| F0_Hard - F0_Veggie | 1.339156 | 0.5843639 | 147 | 2.292 | 0.5268 |
| F0_Hard - F1_Veggie | 1.668353 | 0.5505810 | 147 | 3.030 | 0.1249 |
| F0_Hard - F2_Veggie | 2.719216 | 0.5843639 | 147 | 4.653 | 0.0005 |
| F0_Hard - F3_Veggie | 2.142840 | 0.5651203 | 147 | 3.792 | 0.0131 |
| F1_Hard - F2_Hard | -1.059692 | 0.4802599 | 147 | -2.206 | 0.5882 |
| F1_Hard - F3_Hard | -0.592213 | 0.4695862 | 147 | -1.261 | 0.9884 |
| F1_Hard - F0_Soft | -3.171067 | 0.5240065 | 147 | -6.052 | <.0001 |
| F1_Hard - F1_Soft | -0.308064 | 0.4519841 | 147 | -0.682 | 1.0000 |
| F1_Hard - F2_Soft | 0.256992 | 0.4695862 | 147 | 0.547 | 1.0000 |
| F1_Hard - F3_Soft | 0.358816 | 0.4695862 | 147 | 0.764 | 0.9999 |
| F1_Hard - F0_Veggie | -0.796695 | 0.4925763 | 147 | -1.617 | 0.9218 |
| F1_Hard - F1_Veggie | -0.467498 | 0.4519841 | 147 | -1.034 | 0.9981 |
| F1_Hard - F2_Veggie | 0.583365 | 0.4925763 | 147 | 1.184 | 0.9933 |
| F1_Hard - F3_Veggie | 0.006989 | 0.4695862 | 147 | 0.015 | 1.0000 |
| F2_Hard - F3_Hard | 0.467479 | 0.5034492 | 147 | 0.929 | 0.9993 |
| F2_Hard - F0_Soft | -2.111375 | 0.5545564 | 147 | -3.807 | 0.0125 |
| F2_Hard - F1_Soft | 0.751628 | 0.4870725 | 147 | 1.543 | 0.9435 |
| F2_Hard - F2_Soft | 1.316684 | 0.5034492 | 147 | 2.615 | 0.3109 |
| F2_Hard - F3_Soft | 1.418508 | 0.5034492 | 147 | 2.818 | 0.2057 |
| F2_Hard - F0_Veggie | 0.262997 | 0.5249584 | 147 | 0.501 | 1.0000 |
| F2_Hard - F1_Veggie | 0.592194 | 0.4870725 | 147 | 1.216 | 0.9916 |
| F2_Hard - F2_Veggie | 1.643058 | 0.5249584 | 147 | 3.130 | 0.0968 |
| F2_Hard - F3_Veggie | 1.066682 | 0.5034492 | 147 | 2.119 | 0.6507 |
| F3_Hard - F0_Soft | -2.578854 | 0.5453387 | 147 | -4.729 | 0.0004 |
| F3_Hard - F1_Soft | 0.284149 | 0.4765513 | 147 | 0.596 | 1.0000 |
| F3_Hard - F2_Soft | 0.849205 | 0.4932775 | 147 | 1.722 | 0.8827 |
| F3_Hard - F3_Soft | 0.951029 | 0.4932775 | 147 | 1.928 | 0.7762 |
| F3_Hard - F0_Veggie | -0.204482 | 0.5152115 | 147 | -0.397 | 1.0000 |
| F3_Hard - F1_Veggie | 0.124715 | 0.4765513 | 147 | 0.262 | 1.0000 |
| F3_Hard - F2_Veggie | 1.175578 | 0.5152115 | 147 | 2.282 | 0.5339 |
| F3_Hard - F3_Veggie | 0.599202 | 0.4932775 | 147 | 1.215 | 0.9916 |
| F0_Soft - F1_Soft | 2.863004 | 0.5302573 | 147 | 5.399 | <.0001 |
| F0_Soft - F2_Soft | 3.428059 | 0.5453387 | 147 | 6.286 | <.0001 |
| F0_Soft - F3_Soft | 3.529883 | 0.5453387 | 147 | 6.473 | <.0001 |
| F0_Soft - F0_Veggie | 2.374373 | 0.5652562 | 147 | 4.201 | 0.0031 |
| F0_Soft - F1_Veggie | 2.703570 | 0.5302573 | 147 | 5.099 | 0.0001 |
| F0_Soft - F2_Veggie | 3.754433 | 0.5652562 | 147 | 6.642 | <.0001 |
| F0_Soft - F3_Veggie | 3.178057 | 0.5453387 | 147 | 5.828 | <.0001 |
| F1_Soft - F2_Soft | 0.565056 | 0.4765513 | 147 | 1.186 | 0.9932 |
| F1_Soft - F3_Soft | 0.666880 | 0.4765513 | 147 | 1.399 | 0.9729 |
| F1_Soft - F0_Veggie | -0.488631 | 0.4992208 | 147 | -0.979 | 0.9989 |
| F1_Soft - F1_Veggie | -0.159434 | 0.4592163 | 147 | -0.347 | 1.0000 |
| F1_Soft - F2_Veggie | 0.891429 | 0.4992208 | 147 | 1.786 | 0.8537 |
| F1_Soft - F3_Veggie | 0.315053 | 0.4765513 | 147 | 0.661 | 1.0000 |
| F2_Soft - F3_Soft | 0.101824 | 0.4932775 | 147 | 0.206 | 1.0000 |
| F2_Soft - F0_Veggie | -1.053687 | 0.5152115 | 147 | -2.045 | 0.7014 |
| F2_Soft - F1_Veggie | -0.724490 | 0.4765513 | 147 | -1.520 | 0.9493 |
| F2_Soft - F2_Veggie | 0.326374 | 0.5152115 | 147 | 0.633 | 1.0000 |
| F2_Soft - F3_Veggie | -0.250003 | 0.4932775 | 147 | -0.507 | 1.0000 |
| F3_Soft - F0_Veggie | -1.155511 | 0.5152115 | 147 | -2.243 | 0.5620 |
| F3_Soft - F1_Veggie | -0.826314 | 0.4765513 | 147 | -1.734 | 0.8774 |
| F3_Soft - F2_Veggie | 0.224550 | 0.5152115 | 147 | 0.436 | 1.0000 |
| F3_Soft - F3_Veggie | -0.351827 | 0.4932775 | 147 | -0.713 | 1.0000 |
| F0_Veggie - F1_Veggie | 0.329197 | 0.4992208 | 147 | 0.659 | 1.0000 |
| F0_Veggie - F2_Veggie | 1.380060 | 0.5362491 | 147 | 2.574 | 0.3359 |
| F0_Veggie - F3_Veggie | 0.803684 | 0.5152115 | 147 | 1.560 | 0.9390 |
| F1_Veggie - F2_Veggie | 1.050863 | 0.4992208 | 147 | 2.105 | 0.6603 |
| F1_Veggie - F3_Veggie | 0.474487 | 0.4765513 | 147 | 0.996 | 0.9987 |
| F2_Veggie - F3_Veggie | -0.576376 | 0.5152115 | 147 | -1.119 | 0.9960 |

**Table S9.** Post-hoc Tukey comparisons for age at first reproduction for females

| **repr_age** |  |  |  |
| --- | --- | --- | --- |
| lm(  first_reproduction_age ~ group,  data = data) | | | |
| *Predictors* | *Estimates* | *CI* | *p* |
| (Intercept) | 3.8 | 3.23 – 4.37 | **<0.001** |
| group [F0_Hard] | -0.00 | -0.87 – 0.87 | 1.000 |
| group [F1_Hard] | 0.84 | 0.15 – 1.52 | **0.016** |
| group [F2_Hard] | 0.50 | -0.31 – 1.31 | 0.225 |
| group [F3_Hard] | 0.57 | -0.15 – 1.30 | 0.122 |
| group [F0_Soft] | 0.07 | -0.81 – 0.94 | 0.881 |
| group [F1_Soft] | 1.05 | 0.34 – 1.75 | **0.004** |
| group [F2_Soft] | 0.84 | 0.05 – 1.63 | **0.038** |
| group [F3_Soft] | -0.07 | -1.03 – 0.89 | 0.882 |
| group [F0_Veggie] | -0.04 | -0.88 – 0.81 | 0.935 |
| group [F1_Veggie] | 0.85 | 0.11 – 1.58 | **0.024** |
| group [F2_Veggie] | 1.75 | 0.94 – 2.56 | **<0.001** |
| group [F3_Veggie] | 0.55 | -0.22 – 1.31 | 0.159 |
| Hard |  |  |  |
| **repr_age** |  |  |  |
| *Predictors* | *Estimates* | *CI* | *p* |
| (Intercept) | 3.8 | 3.10 – 4.50 | **<0.001** |
| generation [F1] | 0.84 | 0.04 – 1.64 | **0.041** |
| generation [F2] | 0.50 | -0.42 – 1.42 | 0.286 |
| generation [F3] | 0.57 | -0.27 – 1.42 | 0.181 |
| Soft |  |  |  |
| **repr_age** |  |  |  |
| *Predictors* | *Estimates* | *CI* | *p* |
| (Intercept) | 3.87 | 3.23 – 4.50 | **<0.001** |
| generation [F1] | 0.98 | 0.23 – 1.73 | **0.011** |
| generation [F2] | 0.77 | -0.06 – 1.60 | 0.068 |
| generation [F3] | -0.14 | -1.12 – 0.84 | 0.778 |
| Veggie |  |  |  |
| **repr_age** |  |  |  |
| *Predictors* | *Estimates* | *CI* | *p* |
| (Intercept) | 3.76 | 3.16 – 4.37 | **<0.001** |
| generation [F1] | 0.88 | 0.12 – 1.64 | **0.023** |
| generation [F2] | 1.79 | 0.96 – 2.61 | **<0.001** |
| generation [F3] | 0.58 | -0.20 – 1.36 | 0.143 |

**Table S10.** Results of Cox proportion hazard models for survival

| Comparison with ML |  |  |  |
| --- | --- | --- | --- |
| **Surv(surv_months,natural_d)** |  |  |  |
| **coxph(  Surv(surv_months, natural_d) ~ sex + group, data = surv2_nat)** |  |  |  |
| *Predictors* | *Estimates* | *CI* | *p* |
| sex [m] | 0.90 | 0.71 – 1.16 | 0.427 |
| group [F0.Hard] | 0.80 | 0.40 – 1.61 | 0.533 |
| group [F1.Hard] | 1.51 | 0.83 – 2.76 | 0.178 |
| group [F2.Hard] | 0.91 | 0.51 – 1.62 | 0.739 |
| group [F3.Hard] | 2.01 | 1.08 – 3.75 | **0.027** |
| group [F0.Soft] | 0.56 | 0.27 – 1.15 | 0.113 |
| group [F1.Soft] | 1.08 | 0.59 – 1.99 | 0.804 |
| group [F2.Soft] | 1.24 | 0.68 – 2.25 | 0.484 |
| group [F3.Soft] | 3.8 | 1.99 – 7.23 | **<0.001** |
| group [F0.Veggie] | 0.58 | 0.26 – 1.32 | 0.197 |
| group [F1.Veggie] | 1.43 | 0.79 – 2.58 | 0.237 |
| group [F2.Veggie] | 1.36 | 0.77 – 2.39 | 0.285 |
| group [F3.Veggie] | 3.48 | 1.82 – 6.68 | **<0.001** |
| Hard |  |  |  |
| **Surv(surv_months,** |  |  |  |
| **natural_d)** |  |  |  |
| *Predictors* | *Estimates* | *CI* | *p* |
| generation [F1] | 0.84 | 0.45 – 1.53 | 0.562 |
| generation [F2] | 1.28 | 0.72 – 2.29 | 0.399 |
| generation [F3] | 1.02 | 0.54 – 1.91 | 0.955 |
| Soft |  |  |  |
| **Surv(surv_months,** |  |  |  |
| **natural_d)** |  |  |  |
| *Predictors* | *Estimates* | *CI* | *p* |
| generation [F1] | 1.13 | 0.59 – 2.13 | 0.718 |
| generation [F2] | 1.45 | 0.77 – 2.72 | 0.244 |
| generation [F3] | 1.45 | 0.73 – 2.89 | 0.285 |
| Veggie |  |  |  |
| **Surv(surv_months,** |  |  |  |
| **natural_d)** |  |  |  |
| *Predictors* | *Estimates* | *CI* | *p* |
|  |  |  |  |
| generation [F1] | 2.77 | 1.33 – 5.79 | **0.007** |
| generation [F2] | 2.97 | 1.46 – 6.06 | **0.003** |
| generation [F3] | 1.55 | 0.70 – 3.41 | 0.278 |
